# Novel missense variants in brain morphogenic genes associated with depression and schizophrenia

**DOI:** 10.3389/fpsyt.2024.1338168

**Published:** 2024-04-18

**Authors:** Maxim Karagyaur, Alexandra Primak, Kirill Bozov, Dmitriy Sheleg, Mikhail Arbatsky, Stalik Dzhauari, Maria Illarionova, Ekaterina Semina, Larisa Samokhodskaya, Polina Klimovich, Arkadiy Velichko, Mikhail Drach, Ekaterina Sotskaya, Vladimir Popov, Kseniya Rubina, Mariia Parfenenko, Julia Makus, Boris Tsygankov, Vsevolod Tkachuk, Elena Neyfeld

**Affiliations:** ^1^ Faculty of Medicine, Lomonosov Moscow State University, Moscow, Russia; ^2^ Institute for Regenerative Medicine, Medical Research and Education Center, Lomonosov Moscow State University, Moscow, Russia; ^3^ Federal State Budgetary Educational Institution of the Higher Education “A.I. Yevdokimov Moscow State University of Medicine and Dentistry” of the Ministry of Healthcare of the Russian Federation, Moscow, Russia; ^4^ Medical Research and Education Center, Lomonosov Moscow State University, Moscow, Russia

**Keywords:** paranoid schizophrenia, major depressive disorder, brain morphogenesis, neurotrophines, guidance molecules, NGS (next generation sequencing), ARMS (amplification refractory mutation system)

## Abstract

**Introduction:**

Impaired function of brain morphogenic genes is considered one of the predisposing factors for the manifestation of psychiatric and cognitive disorders, such as paranoid schizophrenia (SCZ) and major depressive disorder (MDD). Identification of such genes (genes of neurotrophic factors and guidance molecules among them) and their deleterious genetic variants serves as a key to diagnosis, prevention, and possibly treatment of such disorders. In this study, we have examined the prevalence of genomic variants in brain morphogenic genes in individuals with SCZ and MDD within a Russian population.

**Methods:**

We have performed whole-exome sequencing of 21 DNA samples: 11 from individuals with SCZ and 10 with MDD, followed by ARMS (Amplification-Refractory Mutation System) based screening of detected single nucleotide variants (SNVs) in larger groups: 102 for individuals with SCZ, 79 for those with MDD and 103 for healthy donors.

**Results:**

Whole-exome sequencing has revealed 226 missense mutations in 79 genes (out of 140 studied), some of which occur in patients with psychiatric disorders significantly more frequently than in healthy donors. We have identified previously undescribed genomic variants in brain morphogenic genes: *CDH2* (rs1944294-T and rs17445840-T), *DCHS2* (rs11935573-G and rs12500437-G/T) and *CDH23* (rs1227051-G/A), significantly associated with the incidence of SCZ and MDD in the Russian population. For some SNVs (rs6265-T, rs1944294-T, rs11935573-G, rs4760-G) sex-biased differences in their prevalence between SCZ/MDD patients and healthy donors was detected.

**Discussion:**

However, the functional significance of the SNVs identified has still to be confirmed in cellular and animal models. Once it is fulfilled, these SNVs have the potential to complement the diagnostic toolbox for assessing susceptibility to mental disorders. The data obtained indirectly confirm the importance of adequate brain structure formation for its correct functioning and preservation of mental health.

## 1 Introduction

Mental and cognitive disorders are a growing concern in modern society, affecting over 1 billion people worldwide in 2022, according to the World Health Organization (1). Understanding the mechanisms underlying susceptibility to mental diseases and developing approaches for timely diagnosis, prevention, and treatment are essential. Genetic factors are increasingly recognized as contributors to the predisposition for most mental disorders (2), and impaired brain morphogenesis is believed to be a significant underlying cause (3, 4).

Brain development is a complex, multistage process that relies on the coordinated actions of various cellular and molecular participants (5, 6). Dysregulation or dysfunction of genes involved in brain development, including those encoding neurotrophic factors, guidance molecules, growth factor receptors, intercellular adhesion molecules, among others, can alter brain structure or wiring, predisposing individuals to mental and cognitive disorders. For example, genomic variants such as rs11030103-G, rs6265-T and rs28722151-G within the brain-derived neurotrophic factor (*BDNF*) gene correlate with an elevated risk of major depressive disorder (MDD) in the Mexican American population (7). Similarly, variants rs2856813-G and rs6678788-T within the nerve growth factor (*NGF*) gene are linked to primary affective disorders in women within the American population (8). In the context of schizophrenia, single nucleotide genomic variants (SNPs) within the genes encoding the receptor tyrosine kinase *ERBB4* (rs707284-G and rs7598440-A) and the guidance molecule *EFNB1* (rs104894803-A and rs104894801-T) have been associated with increased predisposition in Jewish populations (9) and Craniofrontonasal syndrome (a condition characterized by encephalocele and intellectual disability) (10), respectively.

Despite the morphogenetic theory of mental disorders being originally put forth in 1891 (11) and subsequently bolstered by a substantial body of evidence, our comprehension of the specific contributions of individual molecules to shaping human mental activity and mental well-being remains notably limited. This underscores the necessity for more extensive research in this domain. Identifying such genes, elucidating the functional significance of their genetic variants, and developing methods for their early detection and therapeutic intervention are key objectives in the field of molecular psychiatry (12–14). Furthermore, these investigations hold the potential to deepen our comprehension of the roles played by individual molecules in the intricate mechanisms governing brain development and function.

Complete gene knockout mutations are a rarity. Instead, it is far more common to encounter point mutations in the coding and regulatory gene loci. These mutations can impair the activity, expression, compartmentalization, and interprotein interactions of the encoded proteins, thereby potentially undermining their function and the processes they participate in. Based on these data, we formulated a hypothesis: the exploration of brain morphogenic genes, particularly their coding regions, within individuals afflicted by psychiatric disorders could potentially unveil novel genomic variants or even genes linked to susceptibility to mental illnesses.

Recent studies have shown that the functional significance of specific genetic variants in mental illness susceptibility can vary depending on genetic context and environmental factors. This variability is evident in the different effects of certain genetic variants across diverse populations or subgroups, such as gender-specific effects. For example, a dinucleotide polymorphism (CA23) in the A3/147 bp NTF3 promoter region was associated with schizophrenia susceptibility in the Japanese population (15) but not in the American and European Caucasian populations (16–19). Similarly, trinucleotide repeats (AGG)n in the *GDNF* gene were linked to psychiatric disorders in the Japanese population but not in the Italian, English, or Chinese populations (20, 21).

Several studies have investigated the association between specific genetic variants in the Russian population and the incidence of mental illness (22–24). Given the limited number of studies that have examined the possible influence of brain morphogenic genes on an individual’s mental well-being within the Russian population, conducting such research could potentially unveil unique genomic variants specific to this population. Consequently, the findings from this study may help elucidate universal or population-specific mechanisms related to the development and compensation of mental disorders.

Therefore, the primary objective of this study was to identify genetic variants within brain morphogenic genes that may be associated with the occurrence of paranoid schizophrenia and major depressive disorder (MDD) in the Russian population.

## 2 Materials and methods

### 2.1 Subjects

This study was conducted in compliance with the Declaration of Helsinki and received approval from the Inter-University Ethics Committee on December 16, 2021 (protocol no. 11), as shown in **Supplementary Figure 1**. All participants were unrelated Caucasians recruited from Russia, primarily from Moscow and the Moscow region. The diagnosis of schizophrenia or depressive disorder was made by a psychiatrist after a clinical interview following the criteria outlined in the Clinical Guidelines “Schizophrenia” (2019) and “Depressive Episode, Recurrent Depressive Disorder” (2019) issued by the Russian Psychiatric Society (25, 26). Preferably, individuals with a family history of psychiatric conditions were included in the study groups. The diagnostic criteria are detailed in **Supplementary 1**.

The “schizophrenia” group consisted of 102 subjects with a median age of 33 years (26 to 47), comprising 54% men and 46% women. The median duration of illness at the time of the study was 8 years (4 to 17.8), and 25.5% of patients had a family history of psychiatric disorders. In the “depression” group, there were 79 subjects with a median age of 31 years (22.5 to 41), comprising 24% men and 76% women. The median duration of illness at the time of the study was 11 years (7.5 to 18), and 15% of patients had a family history of psychiatric disorders. The median age of disease onset was 23 years (19 to 29.8) in the “schizophrenia” group and 17 years (12 to 29) in the “depression” group. Eight individuals with mental illnesses declined to participate in the study due to concerns about the potential disclosure of their personal information.

Healthy donors in the “control” reference group were included based on the absence of mental illness symptoms, no history of referral to psychiatric care centers, and an unburdened psychiatric family history. The “control” group consisted of 103 healthy donors with a median age of 27 years (24.5 to 31.5), comprising 31.1% men and 68.9% women, with no psychiatric family history. Five healthy donors with a family history of psychiatric disorders were not included in the study. Depersonalized information on study participants is listed in **Supplementary Tables 1-3**.

### 2.2 Next-generation sequencing and data curation

Genomic DNA was extracted from peripheral blood mononuclear cells (PBMCs) obtained from both patients and healthy donors. Approximately 10 ml of whole venous blood was collected in an anticoagulant-containing tube. PBMCs were separated using density gradient centrifugation in Ficoll (density 1.077 g/ml) (Paneco, Russia, #Р053), as previously described (27). Genetic DNA was isolated from PBMCs using the QiAamp DNA Blood Mini Kit (250) (Qiagen, #51106) following the manufacturer’s recommendations. Twenty-one DNA samples from the study groups (11 from “schizophrenia” and 10 from “depression” groups) were selected for whole-exome sequencing. Genetic DNA libraries were prepared and sent to “Genoanalytica” LLC (https://www.genoanalytica.ru/full-exome-sequencing) for whole-exome sequencing. The average number of reads per DNA sample was 80126945 ± 15411143 (n = 21) and the average coverage per genome was 385 ± 74 (n = 21).

Sequencing results were processed using standard bioinformatic approach. The PRINSEQ tool (28) assessed the quality of next-generation sequencing (NGS) data and performed preprocessing of the sequences, including removal of adapter sequences at the 3’-end, elimination of redundant reads, and removal of low-quality reads. Raw sequence reads were aligned to the human reference genome GRCh37.p13/hg19 using BWAMEM v. 0.7.17 (29) and sorted by genetic position using SAMtools rmdup (30). GATK v. 2.20.2 MarkDuplicates (https://broadinstitute.github.io/picard/) marked duplicate reads and merged data from multiple lanes. Short genetic variants were called using GATK v. 4.1.7.0 HaplotypeCaller (31) and then scored using the GATK tool CNNScoreVariants. The default parameters and model were used. The effect of each variant was assessed using snpEff (32), SIFT (33), and PolyPhen-2 (34, 35) utilities, which evaluated pathogenicity and conservation of the identified variants based on data extracted from dbNSFP (36), ClinVar (37, 38), OMIM (39), and HGMD (40) databases.

This study specifically focused on genes associated with brain tissue morphogenesis in the predisposition to the incidence of cognitive and psychiatric disorders. The investigated genes included guidance receptors/molecules genes (*ADIPOR1, CDH1, CDH2, CDH3, CDH4, CDH5, CDH6, CDH7, CDH8, CDH9, CDH10, CDH11, CDH12, CDH13, CDH14, CDH15, CDH16, CDH17, CDH18, CDH19, CDH20, CDH21, CDH22, CDH23, CDH24, CDH25, CDH26, CDH27, CDHR1, CDHR2, CDHR3, CDHR4, CD44, DCC, EPHA1, EPHA2, EPHA3, EPHA4, EPHA5, EPHA6, EPHA7, EPHA8, EPHA9, EPHA10, EPHB1, EPHB2, EPHB3, EPHB4, EPHB5, EPHB6, ERBB2, ERBB3, ERBB4, IL6R, IL6ST*, *ITGA3, ITGAV, ITGB1, NRP1, NRP2, PCDHGA12, PLAUR, PLXNA1, PLXNA2, PLXNA3, PLXNA4, PLXNB1, PLXNB2, PLXNB3, PLXNC1, PLXND1, UNC5A, UNC5B, UNC5C, UNC5D, UNC5H1, UNC5H2, UNC5H3, UNC5H4, UNC5H5)*, ligands genes (*ADIPOQ, CHRD, EFNA1, EFNA2, EFNA3, EFNA4, EFNA5, EFNB1, EFNB2, EFNB3, IL6, NRG1, NRG2, NRG3, NRG4, NTN1, NTN3, NTN4, NTNG1, NTNG2, PLAU, RELN, SHH, SEMA3A, SEMA3B, SEMA3C, SEMA3D, SEMA3E, SEMA3F, SEMA3G, SEMA4A, SEMA4B, SEMA4C, SEMA4D, SEMA4F, SEMA4G, SEMA5A, SEMA5B, SEMA6A, SEMA6B, SEMA6C, SEMA6D, SEMA7A*), genes of neurotrophic factors (*NGF, BDNF, NTF3, GDNF, VEGFA, VEGFB, VEGFC, VEGFD*), and their receptors (*NTRK1, NTRK2, NTRK3, NGFR, GFRA1, GFRA3, RET*), as well as genes of associated molecules (*PLAT, PLG*), totaling 140 genes. Genes related to neurotransmitter synthesis, transport, reception, uptake, and immune system-related genes were not considered in this study, as they had previously demonstrated roles in the predisposition to psychiatric and cognitive disorders (41, 42).

### 2.3 ARMS (amplification-refractory mutation system) for the SNVs detection

This study primarily focused on investigating the prevalence of single nucleotide genetic variants (SNVs) in brain morphogenic genes within the Russian population. Our analysis, conducted through whole-exome sequencing, revealed that a significant majority of the mutations, specifically 99.6% (225 out of 226), in the 140 target genes were SNVs, while larger mutations were less prominent (1 out of 226) in our dataset. To assess the prevalence of identified SNVs in populations affected by schizophrenia or MDD, as well as in the healthy donor population, the ARMS method (Amplification-refractory mutation system) was employed, as previously described (36, 37). Sixteen single-nucleotide genetic variants were selected for the ARMS study: BDNF (rs6265), CDH2 (rs17445840, rs1944294), CDH3 (rs12923655, rs3114409), CDH13 (rs4782724), CDH23 (rs10999947, rs1227051), CDH19/DCHS1 (rs4758443), CDH27/DCHS2 (rs1352714, rs12500437, rs11935573, rs28561984, rs72731014), PLAU (rs2227564), and PLAUR (rs4760). The selection criteria for these genetic variants included their relevance to brain tissue development, published data on their association with psychiatric, cognitive, or neurological disorders, amino acid substitutions leading to stop codons or changes in charge or polarity, and their frequency among sequenced exomes.

**Figure 1:**
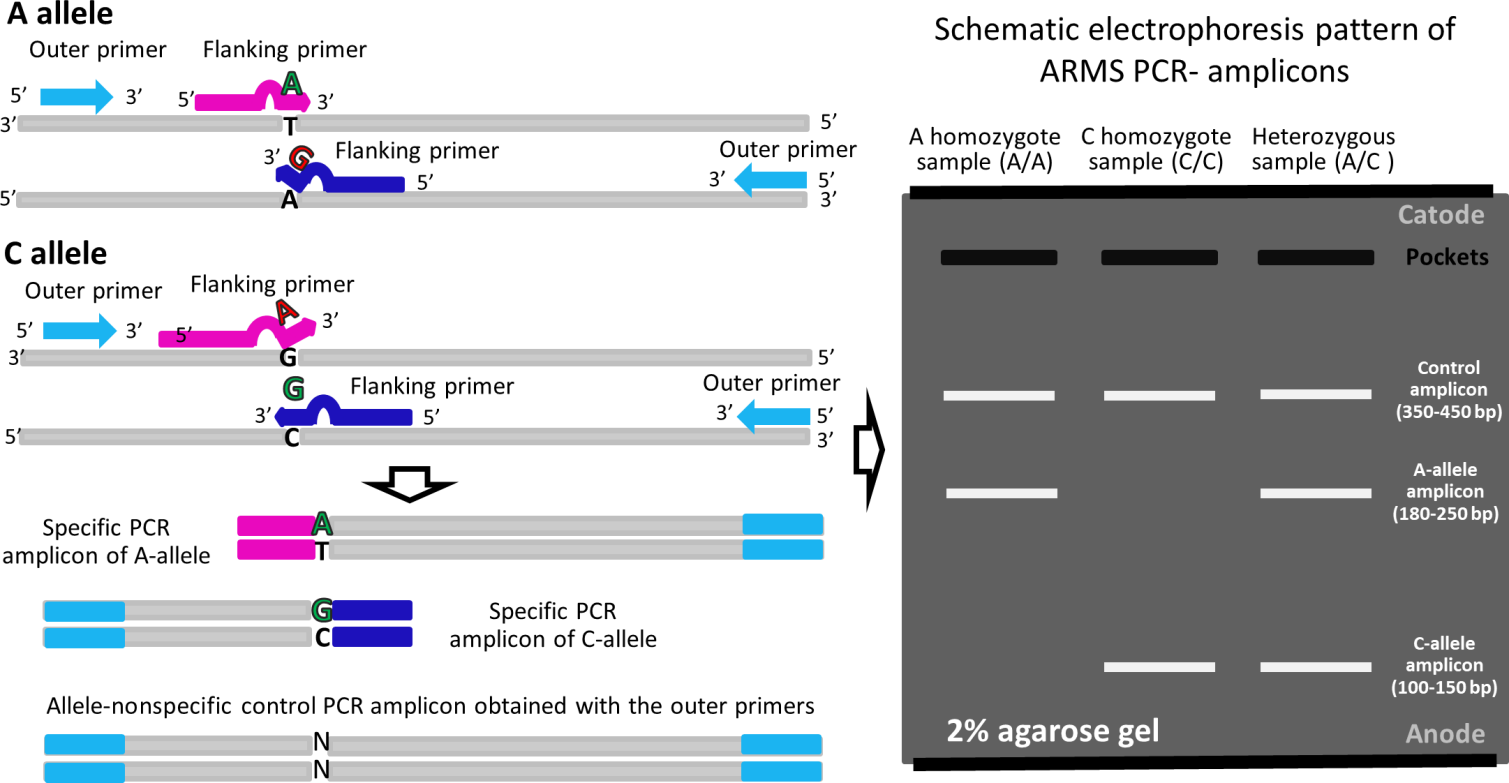
The principles of ARMS (Amplification-refractory mutation system).

Four primers were designed for each studied single nucleotide variant (SNV) as previously described (43, 44): two primers for recognizing different variants of the SNV and two primers flanking the region of interest at a distance of 100-250 base pairs (**Figure 1**). The list of primers and amplification parameters is provided in **Supplementary Table 4**. Amplification was conducted using 5X ScreenMix-HS (UDG) (Evrogen, #PK243L) according to the manufacturer’s recommendations. To visualize the detected genetic variants, amplification products were separated through agarose gel electrophoresis, and the resulting PCR-amplicon patterns were documented using the ChemiDocTM MP imaging system connected to a personal computer. The specificity of allele-recognition inner primers was confirmed by Sanger sequencing of the longest PCR-amplicon generated using the pair of flanking primers for the respective genetic variant (**Figure 1**).

### 2.4 Statistics

Descriptive statistics and the Chi-square test were performed using the SigmaPlot 11.0 program (Systat Software, Inc., Germany). Data were presented as median [25%; 75%]. The incidence of detected alleles (homozygotes AA, BB, and heterozygotes AB) in the experimental (either “schizophrenia” or “depression”) and control groups was calculated for each genetic variant. Pairwise comparisons of the incidence of detected allele variants (AA, BB, or AB) between experimental and control groups were conducted using the “Freeman-Halton extension of the Fisher exact probability test for a two-rows by three-columns contingency table” (45). Given that only two groups were compared at a time, the Bonferroni correction was not applied. Differences were considered significant when p < 0.05.

## 3 Results

### 3.1 Whole-exome sequencing revealed missense mutations in brain morphogenic genes

Whole-exome sequencing of 21 DNA samples obtained from individuals with schizophrenia (11 samples) or MDD (10 samples) and subsequent alignment to the reference human genome GRCh37.p13/hg19 (46) led to the identification of 226 missense mutations in 79 out of 140 studied genes involved in brain tissue morphogenesis and development. Analysis of these amino acid substitutions revealed changes in amino acid radical charge or polarity, such as transitions from polar to nonpolar, positive to negative, and vice versa. These substitutions can potentially disrupt the local charge or polarity of the protein molecule, affecting its structure, compartmentalization, and function. In some instances, we detected premature stop codons (e.g., *CDH2* rs1944294-T (L21Stop) and *NRP2* rs200483574-A (C960Stop)) or frameshift mutations (e.g., SEMA3B gene - rs67324803). A complete list of identified missense mutations in brain morphogenic genes is provided in **Supplementary Table 5**. To improve visualization, we categorized these substitutions based on their potential functional significance: “substitution to a stop codon” (red) > “substitution to an amino acid with opposite charge” ≈ “substitution of a nonpolar amino acid for a polar amino acid” ≈ “substitution of a polar amino acid for a nonpolar amino acid” (orange) > “substitution of a charged amino acid to a polar non-charged one” (yellow) > “amino acid substitution with no change in polarity or charge” (white).

Whole-exome sequencing primarily identified single nucleotide variants (SNVs), comprising a total of 225 out of 226 mutations within the 140 brain morphogenic genes under investigation. Notably, we detected only one larger mutation (rs3062984 TCAT>T (LS>delR)) in the VEGFC gene. Consequently, our study predominantly focused on examining the incidence of SNVs rather than larger mutations within the study groups.

Genes in which no missense mutations were detected in the studied samples are not listed in **Supplementary Table 5**. This list includes the following genes: *ADIPOQ, ADIPOR1, CDH1, CDH6, CDH8, CDH10, CDH14, CDH21, CDH22, CDH25, CDH27, CHRD, EFNA2, EFNA5, EFNB1, EFNB2, EFNB3, EPHA4, EPHA5, EPHA9, EPHA10, EPHB2, EPHB3, EPHB4, EPHB5, ERBB2, ERBB3, ERBB4, GDNF, GFRA1, IL6, IL6R, IL6ST*, *ITGA3, ITGAV, ITGB1, NGFR, NRG1, NRG2, NRG3, NRG4, NTF3, NTN1, NTNG1, NTRK3, PLAT, PLG, PLXNB1, SEMA3F, SEMA4A, SEMA4C, SEMA6B, RELN, RET, SHH, VEGFA, VEGFB, VEGFD, UNC5A, UNC5D, UNC5H1, UNC5H2, UNC5H3, UNC5H4, UNC5H5*.

### 3.2 Screening study revealed significantly different missense mutation prevalence in brain morphogenic genes among “schizophrenia,” “depression,” and healthy volunteers groups

To evaluate the prevalence of specific genetic single-nucleotide variants (SNVs) in larger groups (102 samples for the “schizophrenia” group, 79 samples for the “depression” group, and 103 samples for the healthy volunteers’ group), we selected 16 out of the 226 identified missense mutations that alter the local charge or polarity of protein molecules. These SNVs included: *BDNF* (rs6265), *CDH2* (rs17445840, rs1944294), *CDH3* (rs12923655, rs3114409), *CDH13* (rs4782724), *CDH23* (rs10999947, rs1227051), *CDH19/DCHS1* (rs4758443), *CDH27/DCHS2* (rs1352714, rs12500437, rs11935573, rs28561984, rs72731014), *PLAU* (rs2227564), and *PLAUR* (rs4760). Analysis of these selected genetic variants revealed statistically significant differences in their prevalence between the studied groups (“depression” and “schizophrenia”) and the group of healthy volunteers. For instance, in the “schizophrenia” group, there was a significantly higher occurrence of genetic variants rs1944294-T in the *CDH2* gene (p = 0.0443, n = 102), rs11935573-G (p = 0.0009, n = 102), and rs12500437-G (p = 0.034, n = 102) alleles in the *DCHS2* gene compared to those in the group of healthy volunteers.

In the group of patients with MDD, we found a significantly higher incidence of the SNV rs17445840-T in the *CDH2* gene (p = 0.0315, n = 79) compared to the healthy volunteers. Additionally, there was a significantly increased prevalence of heterozygous variants rs1227051-G/A in the *CDH23* gene (p = 0.0014, n = 79) and rs12500437-G/T in the *DCHS2* gene (p = 0.0390, n = 79) in the “depression” group. Conversely, the incidence of the heterozygous variant rs12923655-A/C in the *CDH3* gene was significantly reduced in this group, with a predominant occurrence of homozygous variants rs12923655-A or rs12923655-C (p = 0.0360, n = 79).

However, the study of the previously described SNP rs6265-T (V66M) in the *BDNF* gene, associated with an increased risk of MDD in the Mexican American population (7) and paranoid schizophrenia in the Chinese population (47–49), revealed no significant difference between the studied groups and the group of healthy volunteers in total selection: for the “schizophrenia” group - p = 0.0641 (n = 102), and for the “depression” group - p = 0.7469 (n = 79). More detailed information on the prevalence of specific missense variants of brain morphogenic genes in experimental and control groups is provided in **Table 1** (genes with statistically significant differences) and **Supplementary Table 6** (other studied genes).

**Table 1:** The incidence of some genetic variants in brain morphogenic genes identified in “schizophrenia” and “depression” (MDD) groups in the Russian population (men and women).

Gene	SNP	Group	N	Var1	Var1/Var2	Var2	P	Allele, %		Χ^2^	P
***CDH2***	**rs1944294 (A>T, L21Stop)**			**A/A**	**A/T**	**T/T**		**A**	**T**		
		Contr	103	71	29	0		171	29		
		SCZ	102	65	30	6	***0.0443***	160	42	*2.32*	*0.128*
		MDD	79	50	28	1	*0.2937*	128	30	*0.986*	*0.321*
	**rs17445840 (C>T, A118T)**			**C/C**	**C/T**	**T/T**		**C**	**T**		
		Contr	103	94	7	0		195	7		
		SCZ	102	91	11	0	*0.4599*	193	11	*0.493*	*0.483*
		MDD	79	67	6	5	***0.0315***	140	16	*5.67*	***0.017***
***CDH3***	**rs12923655 (A>C, T808P)**			**A/A**	**A/C**	**C/C**		**A**	**C**		
		Contr	103	37	41	22		115	85		
		SCZ	102	33	41	27	*0.8340*	107	95	*0.661*	*0.416*
		MDD	79	32	19	28	***0.0360***	83	75	*0.692*	*0.406*
	**rs3114409 (A>C, R778S)**			**A/A**	**A/C**	**C/C**		**A**	**C**		
		Contr	103	54	37	8		145	53		
		SCZ	102	50	42	9	*0.7900*	142	60	*0.293*	*0.589*
		MDD	79	44	31	3	*0.5539*	119	37	*0.282*	*0.595*
***CDH23***	**rs1227051 (G>A, A1575T)**			**G/G**	**G/A**	**A/A**		**G**	**A**		
		Contr	103	9	20	72		38	164		
		SCZ	102	8	33	60	*0.1232*	49	153	*1.465*	*0.226*
		MDD	79	3	35	41	***0.0014***	41	117	*2.237*	*0.135*
	**rs10999947 (G>A, S496N)**			**G/G**	**G/A**	**A/A**		**G**	**A**		
		Contr	103	51	42	7		144	56		
		SCZ	102	57	33	12	*0.2658*	147	57	*0.00954*	*0.922*
		MDD	79	43	31	5	*0.9160*	117	41	*0.0984*	*0.754*
***DCHS2***	**rs12500437 (G>T, P1342H)**			**G/G**	**G/T**	**T/T**		**G**	**T**		
		Contr	103	1	9	90		11	189		
		SCZ	102	4	16	80	*0.1131*	24	176	*4.509*	***0.034***
		MDD	79	0	16	61	***0.0390***	16	138	*2.299*	*0.129*
	**rs72731014 (T>C, T620A)**			**T/T**	**T/C**	**C/C**		**T**	**C**		
		Contr	103	58	35	6		151	47		
		SCZ	102	53	38	10	*0.5552*	144	58	*1.035*	*0.309*
		MDD	79	47	30	2	*0.5689*	124	34	*0.136*	*0.712*
	**rs28561984 (C>T, E2050K)**			**C/C**	**C/T**	**T/T**		**C**	**T**		
		Contr	103	70	28	5		168	38		
		SCZ	102	64	33	5	*0.6886*	161	43	*0.297*	*0.586*
		MDD	79	45	31	2	*0.1595*	121	35	*0.647*	*0.421*
	**rs1352714 (T>C, N1352S)**			**T/T**	**T/C**	**C/C**		**T**	**C**		
		Contr	103	2	3	95		7	193		
		SCZ	102	2	0	100	*0.2897*	4	200	*0.416*	*0.519*
		MDD	79	1	1	77	*0.8463*	3	155	-*	-*
	**rs11935573 (G>A, S1660L)**			**G/G**	**G/A**	**A/A**		**G**	**A**		
		Contr	103	19	69	11		107	91		
		SCZ	102	44	48	9	***0.0009***	136	66	*6.856*	***0.009***
		MDD	79	22	48	7	*0.3453*	92	62	*0.925*	*0.336*

* - Over 20% of the expected values in the contingency table are less than 5. The Chi-square test is inaccurate.

Contr, control; SCZ, Schizophrenia; MDD, major depression disorder.

Italic is used for gene symbols. Bold is used to highlight the statistically significant differences between groups.

In some samples, no allele-specific PCR amplicons were detected on electrophoresis (absence of corresponding bands), despite the presence of a longer PCR amplicon generated with a pair of external flanking primers. Presumably, this phenomenon results from the presence of alternative alleles in the studied genetic variant, which cannot be detected using the predesigned allele-specific internal primers. This discrepancy explains the differences observed between the indicated sample size (N) and the sum of detected homozygous and heterozygous genetic variants (Var1 + Var2 + Var1/Var2) in **Tables 1**–**3** and **Supplementary Tables 6-8**.

**Table 2:** The incidence of some genetic variants in brain morphogenic genes identified in “schizophrenia-female” and “depression-female” (MDD) groups in the Russian population.

Gene	SNP	Group	N	Var1	Var1/Var2	Var2	P	Allele, %		Χ^2^	P
***BDNF***	**rs6265 (C>T, V66M)**			**C/C**	**C/T**	**T/T**		**C**	**T**		
		Contr	71	45	18	0		108	18		
		SCZ	47	38	6	3	***0.0180***	82	12	0.016	0.899
		MDD	60	39	16	2	*0.4510*	94	20	0.264	0.608
***CDH2***	**rs1944294 (A>T, L21Stop)**			**A/A**	**A/T**	**T/T**		**A**	**T**		
		Contr	71	47	21	0		115	21		
		SCZ	47	32	11	4	***0.0495***	75	19	0.580	0.446
		MDD	60	34	25	1	*0.1677*	93	27	1.647	0.199
	**rs17445840 (C>T, A118T)**			**C/C**	**C/T**	**T/T**		**C**	**T**		
		Contr	71	65	5	0		135	5		
		SCZ	47	42	5	0	*0.5199*	89	5	-*	-*
		MDD	60	51	6	3	*0.1473*	108	12	3.381	0.066
***CDH23***	**rs1227051 (G>A, A1575T)**			**G/G**	**G/A**	**A/A**		**G**	**A**		
		Contr	71	8	12	50		28	112		
		SCZ	47	5	19	23	***0.0190***	29	65	3.029	0.082
		MDD	60	2	30	28	***0.0001***	34	86	2.033	0.154
	**rs10999947 (G>A, S496N)**			**G/G**	**G/A**	**A/A**		**G**	**A**		
		Contr	71	34	28	6		96	40		
		SCZ	47	27	14	6	*0.4216*	68	26	0.0197	0.888
		MDD	60	34	22	4	*0.7643*	90	30	0.422	0.516

* - Over 20% of the expected values in the contingency table are less than 5. The Chi-square test is inaccurate.

Contr, control; SCZ, Schizophrenia; MDD, major depression disorder.

Italic is used for gene symbols. Bold is used to highlight the statistically significant differences between groups.

**Table 3:** The incidence of some genetic variants in brain morphogenic genes identified in “schizophrenia-male” and “depression-male” (MDD) groups in the Russian population.

Gene	SNP	Group	N	Var1	Var1/Var2	Var2	P	Allele, %		Χ^2^	P
***CDH3***	**rs12923655 (A>C, T808P)**			**A/A**	**A/C**	**C/C**		**A**	**C**		
		Contr	32	10	17	5		37	27		
		SCZ	55	18	23	13	*0.5668*	59	49	0.0612	*0.805*
		MDD	19	6	5	8	*0.0761*	17	21	1.154	*0.283*
	**rs3114409 (A>C, R778S)**			**A/A**	**A/C**	**C/C**		**A**	**C**		
		Contr	32	20	11	0		51	11		
		SCZ	55	27	23	4	*0.2423*	77	31	1.989	*0.158*
		MDD	19	6	12	0	***0.0433***	24	12	2.276	*0.131*
***DCHS2***	**rs12500437 (G>T, P1342H)**			**G/G**	**G/T**	**T/T**		**G**	**T**		
		Contr	32	0	4	28		4	60		
		SCZ	55	3	6	44	*0.5529*	12	94	0.682	*0.409*
		MDD	19	0	6	12	*0.1381*	6	30	-*	-*
	**rs72731014 (T>C, T620A)**			**T/T**	**T/C**	**C/C**		**T**	**C**		
		Contr	32	13	17	2		43	21		
		SCZ	55	30	16	8	*0.1036*	76	32	0.0708	*0.79*
		MDD	19	9	9	1	*0.8948*	27	11	0.0346	*0.852*
	**rs28561984 (C>T, E2050K)**			**C/C**	**C/T**	**T/T**		**C**	**T**		
		Contr	32	21	8	3		50	14		
		SCZ	55	30	20	5	*0.5259*	80	30	0.371	*0.542*
		MDD	19	8	9	1	*0.1859*	25	11	0.521	*0.47*
	**rs1352714 (T>C, N1352S)**			**T/T**	**T/C**	**C/C**		**T**	**C**		
		Contr	32	0	0	32		0	64		
		SCZ	55	2	0	53	*0.5295*	4	106	-*	-*
		MDD	19	0	0	19	*1.0000*	0	38	-*	-*
	**rs11935573 (G>A, S1660L)**			**G/G**	**G/A**	**A/A**		**G**	**A**		
		Contr	32	4	26	1		34	28		
		SCZ	55	31	19	4	***0.00002***	81	27	6.423	***0.011***
		MDD	19	5	13	1	*0.4384*	23	15	0.122	*0.727*
***PLAUR***	**rs4760 (A>G, L224P)**			**A/A**	**A/G**	**G/G**		**A**	**G**		
		Contr	32	25	7	0		57	7		
		SCZ	55	30	22	3	*0.0664*	82	28	4.441	***0.035***
		MDD	19	10	9	0	*0.0702*	29	9	2.045	*0.153*

* - Over 20% of the expected values in the contingency table are less than 5. The Chi-square test is inaccurate.

Contr, control; SCZ, Schizophrenia; MDD, major depression disorder.

Italic is used for gene symbols. Bold is used to highlight the statistically significant differences between groups.

### 3.3 Screening study revealed sex-biased differences in missense mutation prevalence in brain morphogenic genes

Considering the phenomenon of population- and sex-biased gene expression, the functional significance of certain genetic variants in predisposition to pathological conditions may vary considerably among populations and sexes. This prompted us to assess the prevalence of identified SNVs in male and female subgroups within the experimental and control groups. The “schizophrenia-male” group included 55 male subjects with a median age of 33 [25, 42] years, a median age of manifestation of 22 [18, 28] years, and a median illness duration of 7 [4, 15.5] years (30.9% of men had an aggravated psychiatric family history). The “schizophrenia-female” group included 47 women with a median age of 35 [29, 50] years, a median age of manifestation of 24 [21, 30] years, and a median illness duration of 9 [4.5, 19] years (19.1% of women had an aggravated psychiatric family history). The “depression-male” group included 19 male subjects with a median age of 38 [28.5, 44.5] years, a median age of manifestation of 26 [12, 34] years, and a median illness duration of 13 [9, 18.5] years (5.2% with an aggravated psychiatric family history). The “depression-female” group included 60 women with a median age of 29 [21.8, 36.5] years, a median age of manifestation of 17 [12, 25.5] years, and a median illness duration of 11 [7, 17.3] years (16.7% with an aggravated psychiatric family history). The group of healthy volunteers included 32 men with a median age of 26 [24, 28.3] years and 71 women with a median age of 27 [25, 32.5] years, all with no psychiatric family history.

In the “schizophrenia-female” group, we observed a significantly higher prevalence of the rs6265-T (V66M) variant in the *BDNF* gene (p = 0.0180, n = 47) and rs1944294-T (L21Stop) in the *CDH2* gene (p = 0.0495, n = 47), with a decrease in the corresponding heterozygous variants (**Table 2**). The heterozygous variant rs1227051-G/A in the *CDH23* gene was also found to be more frequent in the “schizophrenia-female” (p = 0.0190, n = 47) and “depression-female” (p = 0.0001, n = 60) groups, with a decrease in the incidence of the homozygous variant rs1227051-A compared to the group of healthy volunteers. Additional information on the prevalence of genetic variants in the “schizophrenia-female” and “depression-female” groups can be found in **Supplementary Table 7**.

In the “schizophrenia-male” group, men exhibited a higher prevalence of the genetic variant rs11935573-G (S1660L) in the *DCHS2* gene (p = 0.00002, n ≥ 32) and the G-allele rs4760 (L224P) in the *PLAUR* gene (p = 0.035, n ≥ 32). In the “depression-male” group, we observed an increased incidence of the heterozygous variant rs3114409-A/C (p = 0.0433, n ≥ 19) in the *CDH3* gene, along with a decrease in the frequency of homozygous variants (**Table 3**). Additional information on the prevalence of genetic variants in the “schizophrenia-male” and “depression-male” groups can be found in **Supplementary Table 8**.

## 4 Discussion

Mental and cognitive disorders pose a significant challenge to modern society (1, 50). Consequently, there is an urgent need to unravel the mechanisms and factors underlying the onset and progression of such disorders. Additionally, developing strategies for early diagnosis, prevention, and treatment is a pressing fundamental and practical objective. Contemporary research indicates that genetic predisposition plays a pivotal role in the pathogenesis of mental and cognitive disorders (2). This predisposition can be established during intrauterine development (3, 4). Several studies have highlighted that altered gene expression or mutations in proteins encoded by specific genes can disrupt brain development and maturation, leading to brain damage and functional imbalances in the brain’s activating and inhibitory systems. Such imbalances can trigger the onset of mental disorders. Given the phenomenon of population-biased gene expression, the significance of specific genetic variants in predisposing individuals to particular diseases may vary across populations. This variability prompted our investigation into the prevalence of genetic variants in brain morphogenic genes within the Russian population suffering from schizophrenia and MDD. Our study was motivated by the scarcity of systematic research in this area, especially within the Russian population.

Here, we have performed whole-exome sequencing of DNA samples obtained from patients suffering from paranoid schizophrenia (11 patients) and MDD (10 patients). Bioinformatic analysis of the sequencing results allowed us to identify 226 missense mutations in 79 genes (out of 140 studied) involved in the processes of neural tissue development and brain morphogenesis. Among the genes studied were those responsible for the development of the nervous system from the neural plate formation (*SHH*, *CHRD* genes) to the establishment of interneuronal connections (neurotrophic factors, guidance molecules) and myelination (*ERBB2-4*, *NRG1-4* genes). Most of the identified missense mutations resulted in the polarity or charge change within a protein molecule, that may impair the expression level, solubility, compartmentalization, and activity of the affected protein. The identified missense polymorphisms were previously described (occurring in the Variation Viewer, ClinVar, etc. databases) (37, 51), however, for the absolute majority of them (except for rs6265 in the *BDNF* gene) (52), no impact on the predisposition to psychiatric diseases (neither by association nor by functional studies) has previously been shown.

In this study, we conducted whole-exome sequencing of DNA samples from individuals diagnosed with paranoid schizophrenia (11 patients) and MDD (10 patients). Bioinformatic analysis of the sequencing data led to the identification of 226 missense mutations in 79 out of 140 genes associated with neural tissue development and brain morphogenesis. These genes encompassed various stages of nervous system development, from neural plate formation (e.g., *SHH*, *CHRD* genes) to the establishment of interneuronal connections (neurotrophic factors, guidance molecules) and myelination (*ERBB2-4*, *NRG1-4* genes). Many of the identified missense mutations resulted in changes in polarity or charge within protein molecules, potentially affecting the expression level, solubility, compartmentalization, and activity of the proteins involved. While most of these missense polymorphisms were previously documented in databases like Variation Viewer and ClinVar (37, 51), none had previously been linked to psychiatric disorders through either association studies or functional research, with the exception of rs6265 in the *BDNF* gene (52).

To gauge the prevalence of certain detected missense genetic variants within the Russian population, we employed the ARMS (Amplification-refractory mutation system) technology (43, 44). We focused on variants within genes related to neurotrophins (*BDNF*), cadherins (*CDH2, CDH3, CDH13, CDH23, CDH19/DCHS1, CDH27/DCHS2*), and the urokinase system (*PLAU* and *PLAUR*). We selected these genes because corresponding missense mutations had been observed in patients with psychiatric disorders. These mutations had the potential to alter the properties of the encoded proteins. Furthermore, literature evidence pointed to the importance of these genes in brain morphogenesis and susceptibility to mental disorders (7, 53–55).

The screening study conducted in patients with paranoid schizophrenia and MDD allowed us to identify several genetic variants within morphogenic genes that exhibited significantly different prevalence between the studied groups and the healthy volunteers. Many of these genetic variants were investigated in the context of mental disorders for the first time. For instance, we discovered that the genetic variants rs1944294-T in the *CDH2* gene and rs11935573-G in the *DCHS2* gene were significantly more common in the “schizophrenia” group than in the healthy volunteers’ group (p < 0.05). These missense mutations result in the formation of a premature stop codon (rs1944294-T -> L21Stop) in one of the *CDH2* isoforms and changes in polarity of the *DCHS2* extracellular domain (rs11935573-G -> S1660L). We hypothesize that these changes may contribute to psychiatric and cognitive disorders. This hypothesis finds indirect support in the literature, where *CDH2* (neural cadherin) is identified as a key molecule in the multipolar-to-bipolar transition and radial migration of postmitotic neurons (7, 48, 49). Mutations in *CDH2* may predispose individuals to attention deficit hyperactivity disorder (ADHD) (56). The *CDH27/DCHS2* gene, according to the literature, plays a role in facial development, and mutations in it are linked to the Cerebro-facio-articular syndrome of Van Maldergem (57, 58). This syndrome is characterized by impaired face and joint development, gray matter dystopia in the brain, and variable neuropsychiatric developmental delays of varying severity.

When we divided subjects within the “schizophrenia” group by sex, we identified additional genetic variants associated with susceptibility to schizophrenia or MDD in the Russian population. Specifically, in the “schizophrenia-female” group, we observed a significantly higher incidence of the rs6265-T (V66M) variant in the *BDNF* gene (p = 0.0180, n = 47) and a lower occurrence of the rs1227051-A variant in the *CDH23* gene (p = 0.0495, n = 47) compared to healthy volunteers. In the “schizophrenia-male” group, there was a higher incidence of the genetic variant rs11935573-G (S1660L) in the *DCHS2* gene (p = 0.00002, n ≥ 32) and the G-allele rs4760 (L224P) in the *PLAUR* gene (p = 0.035, n ≥ 32). These results align with the known roles of *BDNF* and *PLAUR* genes in processes such as neural progenitor proliferation, survival, migration, and the establishment and stabilization of interneuronal connections. Dysfunctions in these genes can manifest as neurological and cognitive deficits. The rs6265-T (V66M) variant in the *BDNF* gene, for instance, reduces inducible BDNF production (59) and is associated with an elevated risk of MDD in the Mexican American population (7) and paranoid schizophrenia in the Chinese population (47, 48). Spatiotemporal perturbations in *PLAUR* expression are presumed to contribute to autism spectrum disorders (60, 61), a notion supported by animal studies (62). However, all previously described SNPs in the *PLAUR* gene associated with susceptibility to psychiatric disorders were located in noncoding regions (61, 63). Similarly, the *CDH23* gene is known to be crucial for the formation of the inner ear hair apparatus (64) and for prepulse inhibition (PPI) in neuronal networks (65, 66). Alterations in CDH23 expression or function may lead to congenital deafness (Usher syndrome) (64) and predisposition to schizophrenia (67). The implication of CDH23 in protein-protein interaction (PPI) processes raises the possibility that detrimental mutations within the *CDH23* gene could potentially contribute to pro-epileptic conditions.

Within the “depression” group, we have identified a notably higher frequency of the genetic variant rs17445840-T in the *CDH2* gene. Additionally, there was an elevated prevalence of heterozygous variants rs1227051-G/A in the *CDH23* gene and rs12500437-G/T in the *DCHS2* gene (p = 0.0014 and p = 0.0390, respectively, n = 79).

*CDH3*, or P-cadherin, although not previously implicated in the pathogenesis of mental disorders, has been shown in several studies to play a critical role in neural cell migration and nerve fiber guidance (68, 69). Dysfunction of *CDH3* may thus lead to impaired brain formation and function. Stratifying the individuals within the “depression” group by gender enabled us to identify a higher occurrence of the heterozygous variant rs3114409-A/C (p = 0.0433, n ≥ 19) in the *CDH3* gene among males. Of note, the *CDH3* gene, also known as P-cadherin, hasn’t been previously associated with the pathogenesis of mental disorders. However, several studies have highlighted its crucial role in neural cell migration and the guidance of nerve fibers (68, 69). Dysfunctions in the *CDH3* gene may potentially lead to disruptions in brain development and function.

It is important to note that the presence of a single genetic variant is rarely sufficient to cause the manifestation of a disease by itself. Instead, the realization of its potential “pathogenicity” typically depends on various contextual factors, including concomitant mutations, expression gene patterns, environmental conditions, and etc. This phenomenon may explain the variable phenotypic expression of such genetic variants and their population- and sex-specific patterns of manifestation (70, 71).

For several genetic variants identified, an association between increased or decreased occurrence of their heterozygous forms and susceptibility to psychiatric disorders has been demonstrated. This could be attributed to mechanisms of overdominance and underdominance, which respectively lead to increased or decreased trait expression in heterozygous individuals (72–74).

Molecular psychiatry, a relatively nascent field, is actively gathering data on the prevalence and functional relevance of specific genomic variants in the development of psychiatric disorders. The discipline is currently in a phase of accumulating knowledge in this regard. When the substantial significance of a particular genomic variant in the pathogenesis of a disease is robustly confirmed through cellular and/or animal models, this knowledge can be harnessed for early disease diagnosis and preventive measures to delay its onset. Moreover, certain genomic variants may serve as potential targets for small molecule drugs or small RNA-based therapeutics, offering a promising avenue to halt the progression of mental illnesses.

In this study, we have identified several genetic variants in brain morphogenic genes that are significantly associated with susceptibility to paranoid schizophrenia or MDD in the Russian population. Many of these genetic variants have not been previously investigated in the context of mental disorders and are documented in the present study for the first time. However, the data we have obtained are insufficient to fully elucidate the mechanisms by which these identified genetic variants affect brain development and function, as well as their contributions to the pathogenesis of psychiatric and cognitive disorders. Unraveling these mechanisms will require additional studies using cellular and animal models, along with genetic technologies (75–77). These future investigations will serve as a continuation of the present study. Upon validation of the functional significance of these genetic variants, they could offer valuable additions to the diagnostic repertoire for evaluating an individual’s susceptibility to mental disorders. Moreover, they might emerge as potential targets in therapeutic strategies aimed at either retarding the progression of mental illnesses or enhancing the effectiveness of antipsychotic pharmacotherapy.

## 5 Limitations of the study

It is important to acknowledge several limitations inherent in this study. First, it should be noted that this research is focused solely on the Russian population, which may limit the broader applicability of our findings. Nevertheless, the significance of the identified molecules (BDNF, CDH2, CDH3, CDH23, DCHS2, PLAUR) in the development and functioning of the human nervous system, along with documented pathogenic mutations in these genes (7, 47–49, 53–64, 67–69), suggests that similar genomic variants disrupting molecular processes may be present in patients with psychiatric disorders from diverse human populations.

Another notable limitation is the relatively small sample size of patients. While this sample size is sufficient for studying the prevalence of individual genomic variants among participants with both schizophrenia and MDD of both sexes, it’s possible that certain less common pathogenic genomic variants have not been identified due to their lower frequency in the population. Additionally, when stratified by gender, the sample sizes become even smaller, preventing certain statistical analyses for a number of genes.

Furthermore, it’s important to acknowledge that this study does not encompass all parameters related to disease onset, progression, treatment response, or hereditary predisposition, as well as the broader genetic context. Additional investigations are needed to determine the predictive value of these genomic variants and their potential to guide treatment strategies within specific populations.

Lastly, this study exclusively focused on the coding (exon) sequences of brain morphogenic genes, without considering intronic and regulatory sequences, which warrant separate investigations.

## Data availability statement

The original contributions presented in the study are included in the article/**Supplementary Material**. Further inquiries can be directed to the corresponding authors.

## Ethics statement

The studies involving humans were approved by Inter-University Ethics Committee, (protocol no. 11, 16 December 2021, http://www.ethicmke.ru/). The studies were conducted in accordance with the local legislation and institutional requirements. Written informed consent for participation in this study was provided by the participants’ legal guardians/next of kin.

## Author contributions

MK: Conceptualization, Data curation, Formal analysis, Funding acquisition, Investigation, Project administration, Resources, Software, Supervision, Validation, Visualization, Writing – original draft, Writing – review & editing. AP: Formal analysis, Investigation, Methodology, Validation, Writing – original draft. KB: Data curation, Formal analysis, Investigation, Methodology, Validation, Visualization, Writing – original draft. DS: Data curation, Formal analysis, Investigation, Methodology, Writing – original draft. MA: Data curation, Formal analysis, Software, Writing – original draft. SD: Data curation, Investigation, Validation, Visualization, Writing – original draft. MI: Data curation, Investigation, Validation, Writing – original draft. ESe: Conceptualization, Formal analysis, Resources, Supervision, Writing – review & editing. LS: Conceptualization, Formal analysis, Methodology, Resources, Supervision, Writing – review & editing. PK: Data curation, Investigation, Methodology, Resources, Supervision, Validation, Writing – original draft. AV: Investigation, Validation, Writing – original draft. MD: Writing – original draft, Investigation, Validation. ESo: Investigation, Writing – original draft. VP: Investigation, Resources, Validation, Writing – original draft. KR: Supervision, Writing – review & editing. MP: Investigation, Writing – original draft. JM: Investigation, Writing – original draft. BT: Conceptualization, Project administration, Resources, Writing – review & editing. VT: Conceptualization, Project administration, Resources, Writing – review & editing. EN: Conceptualization, Investigation, Project administration, Resources, Supervision, Validation, Writing – review & editing.
