# Supplementary material for: Novel missense variants in brain morphogenic genes associated with depression and schizophrenia
Source: Front Psychiatry. 2024 Apr 18;15:1338168. doi: 10.3389/fpsyt.2024.1338168 (PMC11063365; doi:10.3389/fpsyt.2024.1338168)
Supplement: Supplementary file 1 [file DataSheet_1.docx]

Supplementary Material

# Supplementary Data

The diagnosis of major depressive disorder (MDD) was established by a psychiatrist after a clinical interview based on the criteria outlined in the Clinical Guidelines "Depressive Episode, Recurrent Depressive Disorder" (2019) issued by the Russian Psychiatriс Society.

**Diagnostic criteria for the MDD** (Recurrent Depressive Disorder) according to the ICD 10 include:

1. there was at least one mild (F32.0), moderate (F32.1), or severe (F32.2 or .3) depressive episode in the past of at least two weeks in duration separated from the current episode by a period of at least 2 months during which no significant affective symptoms were present;

2. no past episodes meeting the criteria for hypomanic or manic episode (F30.-);

3. the current episode must meet the criteria for a mild, moderate, or severe depressive episode.

**Diagnostic criteria for the MDD** (Depressive Episode) according to the ICD 10 include:

1. a decrease in mood, evident in comparison to the patient's inherent norm, prevalent almost daily and most of the day, that lasts at least two weeks regardless of the situation;

2. the following core symptoms should be present:

- reduced mood;
- a distinct decrease in interest in or enjoyment of activities normally associated with positive emotions;
- decreased energy and increased fatigue;

3. the additional symptoms may present:

- reduced ability to focus and pay attention;
- decreased self-esteem and feelings of insecurity;
- ideas of guilt and humiliation (even in mild depression);
- a gloomy and pessimistic view of the future;
- ideas or actions regarding self-harm or suicide;
- disturbed sleep;
- disturbed appetite.

4. no history of manic/hypomanic symptoms meeting criteria for a manic episode;

5. the episode cannot be attributed to substance use or an organic psychiatric disorder.

The diagnosis of schizophrenia was established by a psychiatrist after a clinical interview based on the criteria outlined in the Clinical Guidelines "Schizophrenia" (2019) issued by the Russian Psychiatriс Society. **Diagnostic criteria for the Schizophrenia** include at least one of the criteria listed in list (1) or at least two features from list (2) must be present for the majority of the psychotic episode lasting at least one month (or for any length of time on most days).

1. a minimum of one of the following criteria:

- "echoing" of thoughts, insertion or withdrawal of thoughts, or openness of thoughts;
- delusions of influence or affect, clearly relating to body or limb movements or to thoughts, actions, or sensations; delusions of perception;
- challucinatory "voices" representing ongoing commentary on the patient's behavior or discussion among themselves, or other types of hallucinatory "voices" emanating from some part of the body;
- persistent delusions of another kind that are culturally inappropriate and completely impossible in content, such as identifying oneself with religious or political figures, claims of superhuman abilities (e.g., the ability to control the weather or communicate with aliens).

2. or at least two of the following criteria:

- chronic hallucinations of any kind if they occur daily for at least one month and are accompanied by delusions (which may be unstable and semiformalized) without distinct affective content;
- neologisms, interruptions in thought resulting in discontinuity or incongruity in speech;
- catatonic behaviors such as agitation, frozen or waxing flexible, negativism, mutism, and stupor;
- "negative" symptoms such as marked apathy, speech impairment, and flattened or inadequate emotional responses (it should be obvious that these are not due to depression or neuroleptic therapy.

**Diagnostic criteria for the** Paranoid schizophrenia (ICD 10 code F20.0):

1. General criteria for schizophrenia should be identified - listed above (ICD 10 codes 20.0- 20.3).

2. Delusions and hallucinations (such as delusions of persecution, importance and relationship, high descent, special mission, bodily alteration, or jealousy; threatening or peremptory "voices", olfactory or gustatory hallucinations, sexual or other bodily sensations).

3. Emotional flattening or inadequacy, catatonic symptoms, or disconnected speech should not dominate the clinical picture, although they may be present to a mild degree of severity.

# Supplementary Figures and Tables

## Supplementary Figures


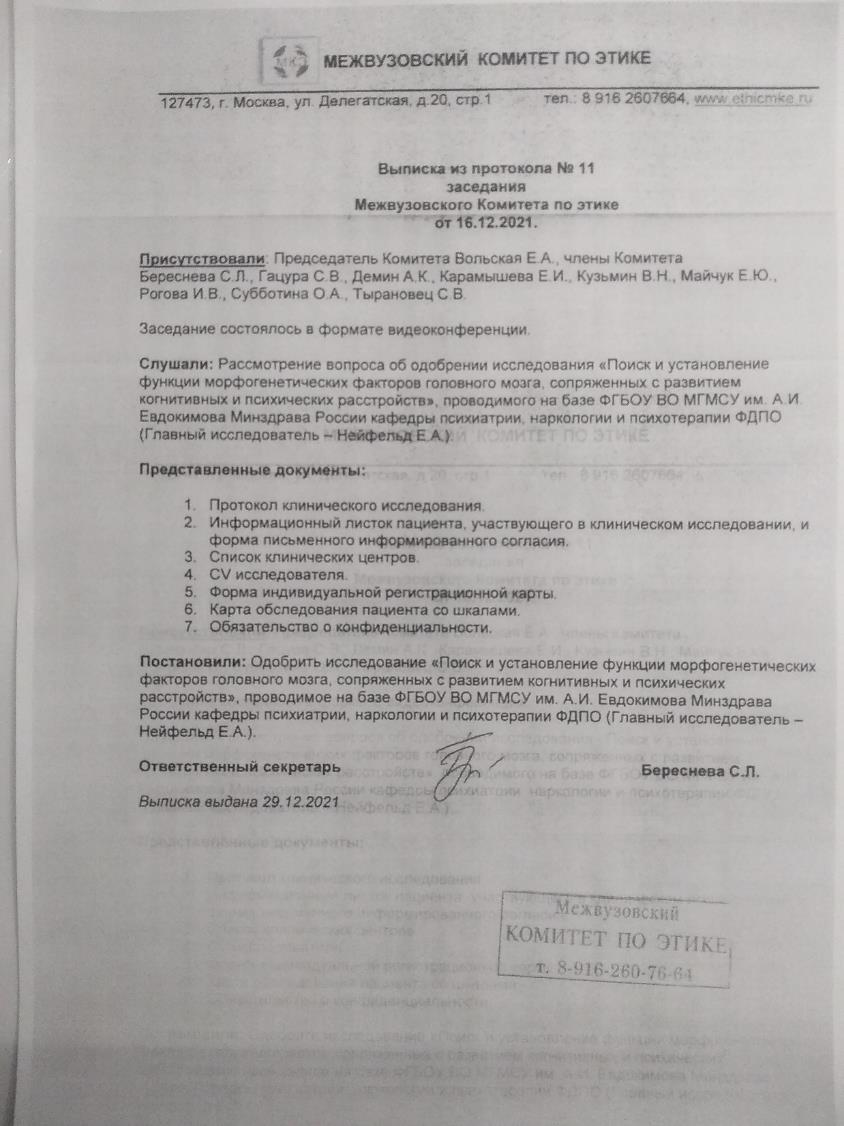


## Supplementary Figure 1. A copy of the research authorization obtained from the Inter-University Ethics Committee on 16.12.2021 (protocol no. 11)

## Supplementary Tables

**Supplementary Table 1.** Detailed information on study participants affected by SCZ

| **Subject #** | **Sex** | **Age, years** | **Diagnosis code (ICD-10)** | **Family history** | **Age of onset, years** | **Disease duration, years** |
| --- | --- | --- | --- | --- | --- | --- |
| S001 | m | 18 | F20.016 | brother's drug addiction | 15 | 3 |
| S002 | m | 53 | F20.016 | - | 30 | 23 |
| S003 | m | 35 | F20.006 | - | 11 | 24 |
| S004 | m | 32 | F20.016 | brother's drug addiction, aunt's gambling addiction | 23 | 9 |
| S005 | m | 55 | F20.016 | - | 35 | 20 |
| S006 | m | 17 | F20.016 | - | 14 | 3 |
| S007 | f | 50 | F20.01 | - | 21 | 29 |
| S008 | f | 43 | F20.01 | - | 28 | 15 |
| S009 | f | 58 | F20.0 | brother's schizophrenia | 22 | 36 |
| S010 | f | 34 | F20.01 | son's autism, sister's alcoholism | 26 | 8 |
| S011 | f | 52 | F20.0 | - | 22 | 30 |
| S012 | m | 26 | F20.016 | - | 23 | 3 |
| S013 | m | 33 | F20.006 | mother's schizophrenia | 21 | 12 |
| S014 | m | 36 | F20.016 | sister's drug addiction | 18 | 18 |
| S015 | m | 51 | F20.016 | mother's schizophrenia | 45 | 6 |
| S016 | m | 58 | F20.0 | - | 37 | 21 |
| S017 | f | 52 | F20.00 | - | 33 | 19 |
| S018 | f | 43 | F20.01 | - | 34 | 9 |
| S019 | f | 52 | F20.016 | - | 36 | 16 |
| S020 | f | 32 | F20.0 | mother's schizophrenia | 25 | 7 |
| S021 | f | 31 | F20.01 | - | 22 | 9 |
| S022 | m | 33 | F20.016 | - | 27 | 6 |
| S023 | m | 23 | F20.004 | brother's schizophrenia | 16 | 7 |
| S024 | m | 31 | F20.016 | - | 28 | 3 |
| S025 | m | 26 | F20.006 | - | 19 | 7 |
| S026 | f | 61 | F20.00 | son's schizophrenia | 30 | 31 |
| S027 | f | 21 | F20.01 | - | 18 | 3 |
| S028 | f | 51 | F20.01 | mother's suicide | 44 | 7 |
| S029 | f | 41 | F20.006 | - | 28 | 13 |
| S030 | f | 45 | F20.01 | - | 41 | 4 |
| S031 | f | 49 | F20.01 | - | 28 | 21 |
| S032 | f | 38 | F20.01 | - | 35 | 3 |
| S033 | f | 44 | F20.01 | sister's parkinsonism | 40 | 4 |
| S034 | f | 22 | F20.01 | - | 20 | 2 |
| S035 | m | 43 | F20.00 | parent's alcoholism | 16 | 27 |
| S036 | m | 35 | F20.004 | - | 13 | 22 |
| S037 | m | 52 | F20.006 | - | 15 | 37 |
| S038 | f | 35 | F20.01 | - | 12 | 23 |
| S039 | f | 57 | F20.01 | - | 32 | 25 |
| S040 | f | 48 | F20.0 | - | 44 | 4 |
| S041 | f | 61 | F20.01 | - | 51 | 10 |
| S042 | f | 30 | F20.0 | - | 26 | 4 |
| S043 | f | 28 | F20.01 | - | 23 | 5 |
| S044 | f | 28 | F20.0 | - | 18 | 10 |
| S045 | m | 26 | F20.0 | grandma's schizophrenia | 22 | 4 |
| S046 | m | 37 | F20.016 | brother's schizophrenia | 27 | 10 |
| S047 | m | 24 | F20.006 | - | 20 | 4 |
| S048 | m | 43 | F20.01 | - | 39 | 4 |
| S049 | m | 24 | F20.016 | - | 22 | 2 |
| S050 | m | 28 | F20.0 | - | 25 | 3 |
| S051 | m | 33 | F20.006 | - | 28 | 5 |
| S052 | m | 35 | F20.016 | - | 23 | 12 |
| S053 | m | 23 | F20.01 | - | 18 | 5 |
| S054 | m | 26 | F20.016 | - | 24 | 2 |
| S055 | f | 31 | F20.01 | - | 28 | 3 |
| S056 | f | 22 | F20.01 | - | 16 | 6 |
| S057 | f | 28 | F23.1 | farther's alcoholism | 23 | 5 |
| S058 | m | 23 | F20.016 | - | 18 | 5 |
| S059 | m | 34 | F20.0 | - | 20 | 14 |
| S060 | m | 47 | F20.004 | - | 37 | 10 |
| S061 | f | 53 | F20.006 | - | 30 | 23 |
| S062 | f | 49 | F20.00 | - | 30 | 19 |
| S063 | f | 55 | F20.00 | - | 25 | 30 |
| S064 | f | 25 | F20.0 | - | 21 | 4 |
| S065 | f | 26 | F20.0 | - | 20 | 6 |
| S066 | f | 52 | F20.0 | - | 46 | 6 |
| S067 | f | 35 | F20.004 | - | 23 | 12 |
| S068 | f | 50 | F20.01 | - | 30 | 20 |
| S069 | f | 27 | F20.01 | grandma's schizophrenia, brother's schizophrenia | 23 | 4 |
| S070 | m | 25 | F20.016 | - | 21 | 4 |
| S071 | m | 28 | F20.016 | aunt's schizophrenia, mom's depression | 23 | 5 |
| S072 | m | 35 | F20.006 | - | 24 | 11 |
| S073 | m | 21 | F20.0 | - | 18 | 3 |
| S074 | f | 25 | F20.01 | - | 20 | 5 |
| S075 | f | 32 | F20.00 | - | 22 | 10 |
| S076 | f | 32 | F20.01 | - | 24 | 8 |
| S077 | f | 21 | F20.01 | - | 17 | 4 |
| S078 | f | 31 | F20.01 | - | 22 | 9 |
| S079 | m | 19 | F20.016 | - | 15 | 4 |
| S080 | m | 23 | F20.0 | - | 19 | 4 |
| S081 | m | 15 | F20.016 | - | 12 | 3 |
| S082 | m | 53 | F20.00 | - | 36 | 17 |
| S083 | m | 47 | F20.006 | - | 39 | 8 |
| S084 | m | 23 | F20.006 | father's schizophrenia | 12 | 11 |
| S085 | f | 30 | F20.01 | - | 22 | 8 |
| S086 | f | 33 | F20.01 | - | 17 | 16 |
| S087 | f | 21 | F20.01 | aunt's schizophrenia, uncle's schizophrenia | 17 | 4 |
| S088 | m | 51 | F20.006 | - | 29 | 22 |
| S089 | m | 38 | F20.0 | - | 30 | 8 |
| S090 | f | 49 | F20.0 | - | 21 | 28 |
| S091 | m | 32 | F20.014 | mother's schizophrenia | 28 | 4 |
| S092 | m | 54 | F21.3 | - | 23 | 31 |
| S093 | m | 45 | F20.0 | grandma's schizophrenia | 35 | 10 |
| S094 | m | 33 | F20.004 | - | 26 | 7 |
| S095 | m | 25 | F20.0 | father's schizophrenia | 18 | 7 |
| S096 | m | 26 | F20.0 | - | 19 | 7 |
| S097 | m | 43 | F20.0 | - | 40 | 3 |
| S098 | m | 24 | F20.0 | mother's alcoholism | 16 | 8 |
| S099 | m | 39 | F20.0 | - | 20 | 19 |
| S100 | m | 28 | F20.0 | brother's schizophrenia | 18 | 10 |
| S101 | m | 29 | F20.0 | grandpa's epilepsy, father's and mother's alcoholism | 10 | 19 |
| S102 | m | 41 | F20.0 | - | 20 | 21 |

**Supplementary Table 2.** Detailed information on study participants affected by MDD

| **Subject #** | **Sex** | **Age, years** | **Diagnosis code (ICD-10)** | **Family history** | **Age of onset, years** | **Disease duration, years** |
| --- | --- | --- | --- | --- | --- | --- |
| D001 | m | 54 | F33.1 | - | 35 | 19 |
| D002 | f | 44 | F41 | - | 29 | 15 |
| D003 | f | 50 | F33.1 | - | 42 | 8 |
| D004 | m | 51 | F41 | - | 33 | 18 |
| D005 | f | 48 | F32 | mother's schizophrenia | 34 | 14 |
| D006 | f | 29 | F33.1 | - | 8 | 21 |
| D007 | m | 34 | F33.1 | - | 11 | 23 |
| D008 | f | 46 | F32 | - | 29 | 17 |
| D009 | f | 41 | F32 | - | 22 | 19 |
| D010 | f | 20 | F33.1 | - | 15 | 5 |
| D011 | f | 27 | F33.1 | - | 11 | 16 |
| D012 | m | 37 | F33.1 | - | 26 | 11 |
| D013 | m | 42 | F33.1 | - | 22 | 20 |
| D014 | f | 31 | F33.1 | - | 11 | 20 |
| D015 | f | 44 | F41 | - | 29 | 15 |
| D016 | f | 35 | F33.1 | - | 17 | 18 |
| D017 | m | 41 | F41 | - | 36 | 5 |
| D018 | f | 27 | F41 | - | 16 | 11 |
| D019 | f | 21 | F33.1 | - | 12 | 9 |
| D020 | f | 28 | F32 | - | 8 | 20 |
| D021 | f | 29 | F41 | - | 18 | 11 |
| D022 | f | 33 | F32 | - | 15 | 18 |
| D023 | f | 20 | F41 | mom's panic attacks | 4 | 16 |
| D024 | m | 23 | F33.1 | - | 8 | 15 |
| D025 | f | 22 | F41 | - | 15 | 7 |
| D026 | f | 19 | F32 | sister's cyclothymia | 2 | 17 |
| D027 | f | 21 | F32 | - | 10 | 11 |
| D028 | m | 21 | F33.1 | mom's depression, grandma's parkinsonism, grandpa's suicide | 10 | 11 |
| D029 | m | 38 | F41 | - | 30 | 8 |
| D030 | f | 21 | F32 | uncle's depression | 10 | 11 |
| D031 | f | 27 | F33.1 | father's alcoholism | 12 | 15 |
| D032 | m | 39 | F33.1 | - | 31 | 8 |
| D033 | f | 19 | F32 | - | 14 | 5 |
| D034 | f | 20 | F32 | - | 15 | 5 |
| D035 | f | 20 | F32 | mother's depression | 13 | 7 |
| D036 | f | 22 | F32 | - | 13 | 9 |
| D037 | f | 20 | F41 | - | 13 | 7 |
| D038 | m | 19 | F32 | - | 5 | 14 |
| D039 | f | 20 | F41 | - | 11 | 9 |
| D040 | f | 19 | F33.1 | - | 14 | 5 |
| D041 | f | 18 | F32 | - | 10 | 8 |
| D042 | f | 20 | F33.1 | mother's schizophrenia | 13 | 7 |
| D043 | f | 36 | F33.1 | - | 12 | 24 |
| D044 | m | 47 | F32 | - | 42 | 5 |
| D045 | f | 28 | F33.1 | - | 17 | 11 |
| D046 | f | 25 | F33.1 | - | 16 | 9 |
| D047 | m | 28 | F41 | - | 12 | 16 |
| D048 | f | 38 | F33.1 | - | 18 | 20 |
| D049 | m | 22 | F32 | - | 12 | 10 |
| D050 | m | 29 | F41 | - | 16 | 13 |
| D051 | f | 36 | F33.9 | mother's depression | 33 | 3 |
| D052 | f | 47 | F32 | - | 33 | 14 |
| D053 | f | 26 | F33.9 | grandpa's parkinsonism | 24 | 2 |
| D054 | f | 44 | F41 | - | 21 | 23 |
| D055 | f | 38 | F32 | - | 30 | 8 |
| D056 | f | 36 | F41 | - | 31 | 5 |
| D057 | f | 41 | F32 | - | 22 | 19 |
| D058 | m | 48 | F33.1 | - | 37 | 11 |
| D059 | m | 32 | F33.1 | - | 13 | 19 |
| D060 | f | 50 | F32 | - | 42 | 8 |
| D061 | f | 35 | F33.1 | - | 25 | 10 |
| D062 | f | 32 | F33.1 | - | 17 | 15 |
| D063 | f | 31 | F41 | - | 27 | 4 |
| D064 | f | 24 | F41 | - | 12 | 12 |
| D065 | f | 28 | F32 | - | 24 | 4 |
| D066 | f | 56 | F33.1 | - | 45 | 11 |
| D067 | f | 29 | F32 | - | 11 | 18 |
| D068 | m | 57 | F41 | - | 33 | 24 |
| D069 | f | 24 | F33.1 | - | 17 | 7 |
| D070 | f | 32 | F32 | - | 23 | 9 |
| D071 | m | 42 | F33.1 | - | 37 | 5 |
| D072 | f | 36 | F41 | - | 18 | 18 |
| D073 | f | 21 | F33.9 | grandpa's and father's alcoholism | 12 | 9 |
| D074 | f | 36 | F33.9 | grandma's and mother's depression | 31 | 5 |
| D075 | f | 35 | F32 | - | 11 | 24 |
| D076 | f | 24 | F41 | - | 18 | 6 |
| D077 | f | 30 | F41 | - | 23 | 7 |
| D078 | f | 54 | F33.1 | - | 32 | 22 |
| D079 | f | 51 | F33.1 | - | 29 | 22 |

**Supplementary Table 3.** Detailed information on healthy donors participated in the study

| **Subject #** | **Sex** | **Age, years** | **Family history** |  | **Subject #** | **Sex** | **Age, years** | **Family history** |
| --- | --- | --- | --- | --- | --- | --- | --- | --- |
| C001 | f | 56 | - |  | C053 | f | 19 | - |
| C002 | f | 31 | - |  | C054 | m | 19 | - |
| C003 | f | 30 | - |  | C055 | f | 23 | - |
| C004 | m | 33 | - |  | C056 | f | 26 | - |
| C005 | f | 28 | - |  | C057 | f | 57 | - |
| C006 | f | 33 | - |  | C058 | f | 28 | - |
| C007 | f | 32 | - |  | C059 | m | 26 | - |
| C008 | f | 27 | - |  | C060 | f | 38 | - |
| C009 | f | 33 | - |  | C061 | f | 36 | - |
| C010 | f | 23 | - |  | C062 | f | 59 | - |
| C011 | f | 23 | - |  | C063 | m | 72 | - |
| C012 | m | 26 | - |  | C064 | f | 53 | - |
| C013 | f | 62 | - |  | C065 | f | 27 | - |
| C014 | f | 19 | - |  | C066 | m | 23 | - |
| C015 | f | 19 | - |  | C067 | f | 23 | - |
| C016 | f | 26 | - |  | C068 | m | 24 | - |
| C017 | f | 32 | - |  | C069 | f | 59 | - |
| C018 | f | 38 | - |  | C070 | m | 28 | - |
| C019 | f | 28 | - |  | C071 | f | 25 | - |
| C020 | f | 26 | - |  | C072 | f | 25 | - |
| C021 | f | 25 | - |  | C073 | m | 24 | - |
| C022 | f | 26 | - |  | C074 | m | 25 | - |
| C023 | f | 25 | - |  | C075 | f | 24 | - |
| C024 | f | 26 | - |  | C076 | f | 22 | - |
| C025 | f | 27 | - |  | C077 | f | 26 | - |
| C026 | m | 26 | - |  | C078 | f | 25 | - |
| C027 | f | 29 | - |  | C079 | m | 24 | - |
| C028 | f | 28 | - |  | C080 | f | 21 | - |
| C029 | f | 28 | - |  | C081 | m | 16 | - |
| C030 | m | 28 | - |  | C082 | f | 22 | - |
| C031 | f | 28 | - |  | C083 | f | 25 | - |
| C032 | f | 28 | - |  | C084 | f | 25 | - |
| C033 | f | 28 | - |  | C085 | f | 39 | - |
| C034 | m | 28 | - |  | C086 | m | 42 | - |
| C035 | f | 28 | - |  | C087 | m | 31 | - |
| C036 | f | 28 | - |  | C088 | f | 38 | - |
| C037 | f | 23 | - |  | C089 | f | 33 | - |
| C038 | f | 23 | - |  | C090 | f | 65 | - |
| C039 | m | 39 | - |  | C091 | m | 26 | - |
| C040 | f | 24 | - |  | C092 | f | 27 | - |
| C041 | f | 21 | - |  | C093 | m | 34 | - |
| C042 | m | 29 | - |  | C094 | f | 27 | - |
| C043 | f | 25 | - |  | C095 | m | 28 | - |
| C044 | m | 26 | - |  | C096 | f | 26 | - |
| C045 | m | 26 | - |  | C097 | m | 27 | - |
| C046 | f | 61 | - |  | C098 | m | 27 | - |
| C047 | f | 48 | - |  | C099 | m | 27 | - |
| C048 | f | 24 | - |  | C100 | m | 26 | - |
| C049 | f | 23 | - |  | C101 | f | 28 | - |
| C050 | f | 39 | - |  | C102 | m | 38 | - |
| C051 | m | 18 | - |  | C103 | m | 21 | - |
| C052 | m | 18 | - |  |  |  |  |  |

**Supplementary Table 4.** Primers and amplification parameters used for ARMS-detection of certain allele variants in brain morphogenic genes

| **Gene** | **Variant ID** | **Primer name** | **Nucleotide sequence** | **Annealing T, ⁰С** | **Amplicons** | **Amplicon length, bp** |
| --- | --- | --- | --- | --- | --- | --- |
| **BDNF** | rs6265 | BDNF_6265_f | ctgcagtctttttgtctgccgcC | 62 | f+r1 -> T-allele (mut) | 300 |
|  |  | BDNF_6265_f1 | CTGGTCCTCATCCAACAGCTCTTCTATCGC |  |  |  |
|  |  | BDNF_6265_r1 | TGACATCATTGGCTGACACTTTCGAACATA |  | f1+r -> С-allele (norm) | 199 |
|  |  | BDNF_6265_r | TATTTCATACTTTGGTTGCATGAAGGCTGC |  |  |  |
| **CDH13** | rs4782724 | CDH13_2724_f | CGGGGTCATTTGTGTTCTTTGTCTCCATGTCCT | 63 | f+r1 -> C-allele (norm) | 166 |
|  |  | CDH13_2724_f1 | GAGAATGGCCCACAGATGCCTGGGCGTGATT |  |  |  |
|  |  | CDH13_2724_r1 | GCAGGCAAATTTACCTGCATGGTTGAAGCTGCAGGTAG |  | f1+r -> T-allele (mut) | 264 |
|  |  | CDH13_2724_r | CTTCAAGTTACATTCAATCCAATTGCCACACAAT |  |  |  |
| **CDH23** | rs10999947 | CDH23_9947_f | TGGGCCAAAGGAGACGTGCGAGAG | 64 | f+r1 -> A-allele (mut) | 170 |
|  |  | CDH23_9947_f1 | GACAATGATGCAGGCACCTTTGGGGAAGTCGG |  |  |  |
|  |  | CDH23_9947_r1 | CTCACCTGTCAGGGTCATCACTGAAGAAGTACT |  | f1+r -> G-allele (norm) | 270 |
|  |  | CDH23_9947_r | GTGCCCCAGAGAGTTGAGGAGACTTGAC |  |  |  |
|  | rs1227051 | CDH23_7051_f | GCTCTCCTCCCAGAACGTGGGTGGAGGTAC | 67 | f+r1 -> A-allele (mut) | 150 |
|  |  | CDH23_7051_f1 | CCTACTACATCACCGAGGGCAACAAGGACATAG |  |  |  |
|  |  | CDH23_7051_r1 | TCTCACCGCTGATGCGGTCCATGCGGAAGCT |  | f1+r -> G-allele (norm) | 280 |
|  |  | CDH23_7051_r | AAGGAGTTGTCAAGGATTCGCCTGCTGTGTG |  |  |  |
| **CDH2** | rs17445840 | CDH2_5840_f | GTGGCCATCCATTAATGTGGTCTGAAGCAAAGC | 57.5 | f+r1 -> T-allele (mut) | 182 |
|  |  | CDH2_5840_f1 | CTCCTCAGTTAAGGTTGGCTTCAGGCTCAATTTTACTAC |  |  |  |
|  |  | CDH2_5840_r1 | CAAGACAAAGAGACCCAGGAAAAGTGGCAAGTTA |  | f1+r -> C-allele (norm) | 315 |
|  |  | CDH2_5840_r | CCTTTAACCTAAGCAGGATATAGGTTTAAGTATTAGGGG |  |  |  |
|  | rs1944294 | CDH2_4294_f | GGTTAACAGAAATTCACATAAGCATTAAATTCCTTG | 59.5 | f+r1 -> T-allele (mut) | 202 |
|  |  | CDH2_4294_f1 | GGAATGAATAAGGCAATTTTTGTTACCTGAAAGGAAAACATAAA |  |  |  |
|  |  | CDH2_4294_r1 | GTGTATCTTCACTGAGAAATTAAAGAACCAAGCAGAACA |  | f1+r -> A-allele (norm) | 308 |
|  |  | CDH2_4294_r | GGGATCAGTGAATCAGATGTAATAAGGGCTCT |  |  |  |
| **CDH3** | rs12923655 | CDH3_3655_f | CTGCAGGTCTCCACCCTGGCAGGAAGC | 64 | f+r1 -> C-allele (mut) | 160 |
|  |  | CDH3_3655_f1 | CCAAGACACAGCCCTTCCACAAATACATGAGGA |  |  |  |
|  |  | CDH3_3655_r1 | GGATGGCTTGTCCACCCCACGTGCTCATAG |  | f1+r -> A-allele (norm) | 246 |
|  |  | CDH3_3655_r | GTCCCACTCCCTCCACTGTCCACAA |  |  |  |
|  | rs3114409 | CDH3_4409_f | CACTTGCTGTCTGCTGGTCCCTGAGTGAATG | 66.5 | f+r1 -> A-allele (norm) | 164 |
|  |  | CDH3_4409_f1 | CAGAGAGGAAATGGAGGCTTGCAGCTGGCAATC |  |  |  |
|  |  | CDH3_4409_r1 | ACAGGTAGTTAGGAGCGGCGGGTCCTGCTT |  | f1+r -> C-allele (mut) | 241 |
|  |  | CDH3_4409_r | CGGCTGCCCCACTCGTTCAGATAATCGTAATC |  |  |  |
| **DCHS1** | rs4758443 | DCHS1_8443_f | GGCCACGCGGATTTCACCAGTGTAAGAGTC | 66.5 | f+r1 -> G-allele (norm) | 257 |
|  |  | DCHS1_8443_f1 | GGTGGGTGCATGGTCATTGACATCGCGCACTA |  |  |  |
|  |  | DCHS1_8443_r1 | GGCCCCTAAGCACCACAGTGTCTGTCACCATCTC |  | f1+r -> A-allele (mut) | 143 |
|  |  | DCHS1_8443_r | GCACAGCTGCAGCCTTGGACAGAGAACAG |  |  |  |
| **DCHS2** | rs1352714 | DCHS2_2714_f | CTTCCATATTTTATGGTGACAACATTACCTGAATATTAACAAC | 55 | f+r1 -> C-allele (mut) | 201 |
|  |  | DCHS2_2714_f1 | GGAAGGTCTATAATCATACGAAAGTATTGTGGTTGTTCTTATTTCACCGT |  |  |  |
|  |  | DCHS2_2714_r1 | TGTGTGTAGAAGATAGTTCTGATCACTTTAAGATTGACGCCAACCG |  | f1+r -> T-allele (norm) | 319 |
|  |  | DCHS2_2714_r | GACTCGTGAAAGTTTATTATACCATTTTGTCCACTTTTTTGTATGC |  |  |  |
|  | rs12500437 | DCHS2_0437_f | tgccacccatCTCTCTATTTGTCCTTGCA | 58.5 | f+r1 -> T-allele (mut) | 311 |
|  |  | DCHS2_0437_f1 | CTTCCCAAATGCTGTTTTTCCCTTCAGAGGCATCG |  |  |  |
|  |  | DCHS2_0437_r1 | GGGTAACAGAAAGAGACTTCATCTTTTTTTTTTTTTTTGGCAGCTAGATA |  | f1+r -> G-allele (norm) | 164 |
|  |  | DCHS2_0437_r | GAGGCCAAGGTGTGAGGATAGCTTGC |  |  |  |
|  | rs11935573 | DCHS2_5573_f | GGCTGGATGGAAGGGAAAATGGGAG | 57 | f+r1 -> A-allele (mut) | 280 |
|  |  | DCHS2_5573_f1 | GATGACTCATCTAGCATAAACGTCATGTTTTCATTTCCCG |  |  |  |
|  |  | DCHS2_5573_r1 | AAGGAAGGAATGGAAAAGTAACATACAGCATCCTCGT |  | f1+r -> G-allele (norm) | 175 |
|  |  | DCHS2_5573_r | ACCACAACCCCACTTTTATTTCTTTCCCCAATG |  |  |  |
|  | rs28561984 | DCHS2_1984_f | CAATAGAAAACATTGACTGGGTCTCTGCAAAACTAAAAAC | 57 | f+r1 -> T-allele (mut) | 145 |
|  |  | DCHS2_1984_f1 | ACAATGACAGTTGTCTGGTTTGTAGGCGACCC |  |  |  |
|  |  | DCHS2_1984_r1 | GGAACAGAACCCTTTTGATGTGTTTCTTTCCCCAA |  | f1+r -> C-allele (norm) | 265 |
|  |  | DCHS2_1984_r | TGACTGATGAGGCTTCTGGTGCATTCAC |  |  |  |
|  | rs72731014 | DCHS2_1014_f | AGAAGATGGGCTCATTGTCATTCACATCATCTACG | 59 | f+r1 -> C-allele (mut) | 167 |
|  |  | DCHS2_1014_f1 | CTCCACCGCCTCCTGGACCTCTCGGTCTAGAAT |  |  |  |
|  |  | DCHS2_1014_r1 | TGATTCCGAAAGCGGTGCGATCAGCACTATCCGTG |  | f1+r -> T-allele (norm) | 302 |
|  |  | DCHS2_1014_r | CTGACCTCAATGACCAACCACCTCTCTTCAG |  |  |  |
| **PLAU** | rs2227564 | PLAU_7564_f | TGAGGGGAGGAGGCAGGGAAGGC | 64 | f+r1 -> C-allele (mut) | 151 |
|  |  | PLAU_7564_f1 | CTGGTGCTATGTGCAGGTGGGCCTAAAGTT |  |  |  |
|  |  | PLAU_7564_r1 | AGTCATGCACCATGCACTCTTGGACAAGTG |  | f1+r -> T-allele (norm) | 256 |
|  |  | PLAU_7564_r | AATTCTTCTGGAGGAGAGGAGGGCTTTTTTC |  |  |  |
| **PLAUR** | rs4760 | PLAUR_4760_f | CACTGGCCTGAGGTCACACAGCAAGTCTGTAG | 62.5 | f+r1 -> G-allele (mut) | 187 |
|  |  | PLAUR_4760_f1 | CAGTCTGGCAGTCATTAGCAGGGTGATGGTAA |  |  |  |
|  |  | PLAUR_4760_r1 | CTCAGCCTGGCCCTGCCCATCTCAGCAC |  | f1+r -> A-allele (norm) | 267 |
|  |  | PLAUR_4760_r | CCAGGAGCTGGAAGTCTCACTCCGTCTTCTC |  |  |  |

**Supplementary Table 5**. The missense SNVs in brain morphogenic genes identified by NGS in DNA samples from the “schizophrenia” and “depression” (MDD )groups.

| **Gene** | **Variant ID** | **Nucleot**  **Subst** | **AA Subst** | **Schizophrenia** | | | | | | | | | | |  | **MDD** | | | | | | | | | |
| --- | --- | --- | --- | --- | --- | --- | --- | --- | --- | --- | --- | --- | --- | --- | --- | --- | --- | --- | --- | --- | --- | --- | --- | --- | --- |
|  |  |  |  | **013** | **020** | **022** | **023** | **024** | **034** | **036** | **060** | **064** | **065** | **069** |  | **003** | **005** | **006** | **007** | **008** | **009** | **010** | **011** | **012** | **013** |
| **BDNF** | rs6265 | C>T | V66M | C | C | C | C | C | C | C | C | C | C | T |  | C | C | C | T | C | C | T | C | C | C |
| **CD44** | rs1071695 | C>G | H85Q | G | C | C | C | C | C | C | C | C | C | C |  | C | C | C | C | C | C | C | C | C | C |
|  | rs9666607 | A>G | K418R | G | G | A | G | G | G | G | G | G | G | A |  | G | G | G | G | G | G | A | G | G | G |
|  | rs1467558 | T>C | I479T | C | C | C | C | C | C | C | C | C | C | C |  | C | C | C | C | C | C | C | C | C | C |
|  | rs140510483 | A>G | I457V | A | A | A | A | G | A | A | A | A | A | A |  | A | A | A | A | A | A | A | A | A | A |
|  | rs11607491 | C>T | T393M | C | C | C | C | C | C | C | T | C | C | C |  | C | C | C | C | C | C | C | C | C | C |
| **CDH11** | rs35213 | A>C | S373A | C | C | C | C | A | C | C | C | C | C | C |  | C | C | C | C | A | C | C | C | C | C |
|  | rs141063325 | G>A | A475V | G | A | G | G | G | G | G | G | G | G | G |  | G | G | G | G | G | G | G | G | A | G |
|  | rs76181686 | C>G | V347L | C | G | C | C | C | C | C | G | C | C | C |  | C | C | G | C | C | C | C | C | C | C |
|  | rs1130821 | C>T | M275I | C | T | C | C | C | C | C | C | T | T | C |  | T | C | C | C | C | C | C | T | T | C |
|  | rs35195 | G>A | T255M | G | G | A | A | G | G | A | G | G | G | A |  | G | G | G | A | G | A | A | G | G | A |
| **CDH12** | rs4371716 | C>T | V68M | T | T | T | T | C | T | T | C | T | C | C |  | T | C | T | T | C | C | T | T | T | T |
|  | rs17328673 | T>C | I284V | T | T | T | T | T | T | T | C | T | T | T |  | T | C | T | T | T | T | T | C | T | T |
| **CDH13** | rs4782724 | C>T | P75S | T | T | T | T | T | T | T | T | T | T | T |  | T | T | T | T | T | T | T | T | C | T |
|  | rs200000145 | G>C | E367Q | G | G | G | G | G | G | G | G | G | G | G |  | C | G | G | G | G | G | G | G | G | G |
| **CDH15** | rs75791347 | A>C | K584Q | A | C | C | C | A | C | A | C | C | C | A |  | A | C | A | A | C | A | A | C | C | A |
|  | rs2287359 | G>T | A37S | G | G | G | G | G | G | G | T | G | G | G |  | G | G | G | G | G | G | G | T | G | G |
| **CDH16** | rs61735427 | C>T | A549T | C | C | C | C | T | C | C | C | C | C | C |  | C | C | C | C | C | C | C | C | C | C |
|  | rs2271023 | G>A | H257Y | G | G | G | G | A | G | G | G | G | G | G |  | G | G | G | G | G | G | G | G | G | G |
| **CDH17** | rs1051624 | T>G | E739A | T | G | G | G | G | G | G | G | T | G | G |  | G | G | G | G | G | G | G | G | G | G |
|  | rs1051623 | C>G | E734D | C | G | G | G | G | G | G | G | G | G | G |  | G | G | G | G | G | G | G | G | G | G |
|  | rs2243518 | T>C | K115E | T | C | C | C | C | C | C | T | C | C | C |  | C | T | C | C | C | T | C | C | C | C |
|  | rs139589295 | T>A | E652V | T | T | T | T | T | T | T | T | T | T | T |  | T | T | T | T | T | T | T | T | T | A |
| **CDH18** | rs12187552 | T>A | Q203H | A | T | T | T | T | T | T | T | T | T | T |  | T | T | T | A | T | T | T | T | T | T |
| **CDH19** | rs72954429 | C>T | V391M | C | C | C | T | C | T | C | T | C | C | T |  | C | C | C | C | C | C | C | C | T | T |
|  | rs55874520 | C>G | G645A | C | C | C | C | C | C | C | C | C | C | C |  | C | C | G | C | C | C | C | C | C | C |
| **CDH2** | rs17445840 | C>T | A118T | C | C | T | C | C | T | C | C | C | C | C |  | C | C | C | T | C | C | C | C | T | C |
|  | rs1944294 | A>T | L>Stop | A | A | A | A | T | A | A | A | A | T | A |  | A | A | T | A | A | A | A | A | A | A |
|  | rs2289664 | T>C | N845S | T | T | T | T | T | T | C | T | T | T | C |  | T | T | T | T | T | T | T | T | T | T |
| **CDH20** | rs1943330 | C>A | P328H | C | C | C | C | C | C | C | C | A | C | C |  | C | C | C | C | C | C | C | C | C | C |
| **CDH23** | rs10999947 | G>A | S496N | A | G | A | A | A | G | G | A | G | G | A |  | G | G | G | G | G | G | G | A | A | A |
|  | rs1227065 | A>G | N1351D | A | G | A | G | G | G | G | G | G | G | G |  | G | G | G | G | A | G | G | G | G | G |
|  | rs1227051 | G>A | A1575T | G | A | G | A | A | A | A | A | A | G | A |  | A | A | A | A | G | A | A | A | A | A |
|  | rs17712523 | G>A | V1675I | G | A | A | A | G | A | G | A | A | G | G |  | G | G | G | G | G | G | A | G | G | G |
|  | rs11592462 | C>G | T1999S | C | G | G | G | G | G | C | G | G | G | G |  | G | G | G | C | G | G | G | C | G | C |
|  | rs1227049 | G>C | G490A | G | G | C | G | G | G | G | G | G | G | G |  | G | G | C | C | G | G | G | G | G | G |
|  | rs3802711 | G>A | R1804Q | G | G | A | A | G | G | A | G | G | G | G |  | A | G | G | G | G | G | G | G | G | G |
|  | rs10466026 | G>A | E2044K | G | G | A | G | G | G | G | G | G | G | G |  | A | G | G | G | G | A | G | A | G | G |
|  | rs4747194 | G>A | R2358Q | G | G | A | A | G | G | A | G | G | G | G |  | A | G | G | G | G | A | G | A | G | G |
|  | rs4747195 | C>T | P2380L | C | C | T | T | C | C | T | C | C | C | C |  | T | C | C | C | C | T | C | T | C | C |
|  | rs56181447 | G>A | R1437Q | G | G | G | G | G | G | G | G | A | G | G |  | G | G | G | A | G | G | G | G | G | G |
|  | rs190672679 | G>A | V2834I | G | G | G | G | G | G | G | G | G | A | G |  | G | G | G | G | G | G | G | G | G | G |
|  | rs143282422 | G>A | A366T | G | G | G | G | G | G | G | G | G | G | G |  | G | G | A | G | G | G | G | G | G | G |
|  | rs41281334 | G>A | V2283I | G | G | G | G | G | G | G | G | G | G | G |  | G | G | G | G | G | A | G | A | G | G |
|  | rs41281316 | G>A | A1222T | G | G | G | G | G | G | G | G | G | G | G |  | G | G | G | G | G | G | A | G | G | G |
|  | rs45583140 | T>C | F3125L | T | T | T | T | T | T | T | T | T | T | T |  | T | T | T | T | T | T | T | C | T | T |
|  | rs2290021 | G>A | T3291M | G | G | G | G | G | G | G | G | G | G | G |  | G | G | G | G | G | G | G | A | G | G |
| **CDH24** | rs377064007 | G>C | L392V | G | G | G | G | C | G | G | G | G | G | G |  | G | G | G | G | G | G | G | G | G | G |
| **CDH26** | rs11086690 | C>G | R301G | C | C | G | C | C | C | C | C | C | C | C |  | C | C | C | C | C | C | C | C | C | C |
|  | rs28409250 | A>G | N404S | A | A | G | A | A | A | A | A | A | A | A |  | A | A | A | A | A | A | A | A | A | A |
|  | rs41310817 | A>G | Q711R | A | A | G | A | A | A | A | A | A | A | A |  | A | A | A | A | A | A | A | A | A | A |
| **CDH3** | rs1126933 | G>C | Q563H | G | C | C | G | C | C | C | C | C | G | G |  | C | C | C | G | G | G | C | G | C | G |
|  | rs12923655 | A>C | T808P | A | C | C | A | C | C | C | C | C | A | C |  | C | C | C | A | A | C | C | A | C | C |
|  | rs3114409 | A>C | R778S | A | A | C | C | A | A | A | A | A | A | A |  | A | A | A | C | A | C | C | A | A | A |
|  | rs34494880 | G>A | R477H | G | G | G | G | G | G | G | A | G | G | G |  | G | G | G | G | G | G | A | G | G | G |
|  | rs2296405 | T>A | N542K | T | T | T | T | T | T | T | T | T | T | A |  | T | T | T | T | T | T | T | T | T | T |
|  | rs34394404 | G>A | V429I | G | G | G | G | G | G | G | G | G | G | G |  | G | G | G | G | G | A | G | G | G | G |
|  | rs2274239 | G>C | K652N | G | G | G | G | G | G | G | G | G | G | G |  | G | G | G | G | G | G | C | G | G | G |
| **CDH4** | rs6142884 | A>G | K625R | G | G | G | G | A | A | A | A | G | A | A |  | G | G | A | G | G | G | G | G | G | G |
|  | rs2427240 | C>G | D446E | C | C | C | G | C | C | C | C | C | C | C |  | C | C | C | C | C | C | C | C | C | C |
|  | rs34937312 | C>T | A141V | C | C | C | C | C | C | C | C | C | T | C |  | C | C | C | C | C | C | C | C | C | C |
| **CDH5** | rs1049970 | T>C | I517T | C | C | C | C | C | C | C | C | C | C | C |  | C | C | C | C | T | C | C | C | C | T |
|  | rs3826229 | C>G | I517M | G | C | C | C | G | C | C | C | C | C | G |  | C | C | C | G | C | C | C | C | C | C |
|  | rs16956504 | T>C | I503T | T | T | T | T | T | C | T | T | T | T | T |  | T | T | T | T | T | T | T | T | T | T |
|  | rs141532757 | G>A | G669S | G | G | G | G | G | G | G | G | G | G | G |  | G | G | G | G | G | G | G | A | G | G |
| **CDH7** | rs2291343 | A>G | N576S | G | G | A | G | G | G | A | G | A | G | G |  | G | G | G | G | A | G | G | A | G | G |
|  | rs2306675 | T>G | D370E | T | T | T | T | T | T | T | T | T | T | T |  | T | T | T | G | T | T | T | T | T | T |
| **CDH9** | rs2288466 | G>A | A38V | A | A | G | A | G | A | G | A | A | G | A |  | A | A | A | A | G | A | A | A | A | A |
|  | rs2288467 | T>C | Y6C | C | C | T | C | T | C | T | C | C | T | C |  | C | C | C | C | T | C | C | C | C | C |
|  | rs34490509 | T>C | E603G | T | T | T | T | T | T | T | T | T | C | T |  | T | T | T | T | T | T | T | T | T | T |
| **CDHR1** | rs12781048 | C>A | H53Q | C | C | C | A | C | C | C | C | C | C | C |  | C | C | C | A | C | A | C | C | C | C |
|  | rs4933975 | A>G | Q217R | A | A | A | A | A | A | A | A | G | A | A |  | A | A | A | A | A | A | A | A | A | A |
|  | rs45584033 | C>T | P812S | C | C | C | C | C | C | C | C | C | C | C |  | C | C | C | C | C | C | T | T | C | C |
| **CDHR2** | rs11134982 | T>C | V424A | C | C | C | C | C | C | C | C | C | C | C |  | C | T | C | C | C | C | C | C | C | C |
|  | rs2291442 | C>T | T1128M | C | T | C | T | C | C | C | T | C | C | C |  | T | T | C | C | C | T | C | C | C | C |
|  | rs752138 | T>C | L766P | T | T | C | T | C | T | C | T | T | T | T |  | T | T | T | T | T | T | T | T | T | T |
|  | rs17078347 | C>A | L1164M | C | C | A | C | C | C | A | C | A | A | C |  | A | C | A | C | C | C | A | C | A | A |
|  | rs115050587 | C>G | A721G | C | C | C | C | C | C | C | C | C | C | G |  | C | C | C | C | C | C | C | C | C | C |
| **CDHR3** | rs6967330 | G>A | C529Y | A | G | G | G | G | G | A | G | G | G | G |  | G | A | G | G | G | G | G | A | G | G |
|  | rs117406926 | C>A | P752T | C | C | C | A | C | C | C | C | C | C | C |  | C | C | C | C | C | C | C | C | C | C |
|  | rs35008315 | G>A | V55M | G | G | G | G | G | G | A | A | A | G | G |  | G | A | G | G | G | A | A | G | G | A |
|  | rs34426483 | G>C | Q61H | G | G | G | G | G | G | C | C | C | G | G |  | G | C | G | G | G | C | C | G | G | C |
|  | rs73195662 | C>G | T532S | C | C | C | C | C | C | C | C | C | C | C |  | C | C | C | C | C | C | C | G | C | C |
| **CDHR4** | rs73079003 | G>A | T291M | G | A | A | A | G | G | G | A | A | G | G |  | G | G | G | G | A | G | A | G | G | G |
| **DCC** | rs9951523 | T>C | F23L | C | C | C | C | C | C | C | C | C | C | C |  | C | C | C | C | C | C | C | C | C | C |
|  | rs2229080 | C>G | R201G | G | C | G | C | G | G | C | C | C | G | G |  | C | G | C | C | G | C | G | C | C | C |
|  | rs144623089 | A>G | K419R | A | A | A | A | A | A | A | A | A | A | A |  | A | A | A | A | A | A | A | G | A | A |
| **DCHS1** | rs4758443 | G>A | T1949M | G | A | A | A | A | A | G | G | A | A | G |  | A | A | G | A | A | A | A | G | A | A |
|  | rs148791938 | G>A | L1835F | G | A | G | G | G | G | G | G | G | G | G |  | G | G | G | A | G | G | G | G | G | G |
|  | rs35599968 | C>G | R2827P | C | C | C | G | C | C | C | C | C | C | C |  | C | C | C | C | G | C | C | C | C | C |
|  | rs117368891 | G>T | S415R | G | G | G | G | G | G | G | G | T | G | G |  | T | G | G | G | G | G | G | G | G | G |
| **DCHS2** | rs1352714 | T>C | N1352S | C | C | C | C | C | C | C | C | C | C | C |  | C | C | C | C | C | C | T | C | C | C |
|  | rs12500437 | G>T | P1342H | T | T | T | T | T | T | T | T | T | T | T |  | T | T | T | T | T | T | T | T | G | T |
|  | rs10017772 | T>C | H768R | C | C | C | C | T | C | C | T | C | C | T |  | C | C | C | C | C | C | C | T | C | C |
|  | rs17373860 | G>A | P209S | A | G | G | G | G | A | A | G | G | G | G |  | G | G | G | G | G | G | G | G | G | G |
|  | rs17373874 | A>C | V190G | C | A | C | C | C | C | A | C | C | C | C |  | A | C | C | C | A | C | C | C | C | C |
|  | rs13149269 | C>T | R15Q | T | C | T | T | T | T | C | T | T | T | T |  | C | T | T | T | C | T | T | T | T | T |
|  | rs11935573 | G>A | S1660L | G | A | G | A | A | A | G | G | A | A | A |  | G | A | A | A | G | A | A | A | A | G |
|  | rs28561984 | C>T | E2050K | C | C | T | C | C | C | T | T | C | C | T |  | C | C | C | C | T | C | C | C | C | T |
|  | rs17031394 | G>C | T1935R | G | G | C | G | G | G | C | C | G | G | C |  | G | G | G | G | C | G | G | G | G | C |
|  | rs61741046 | G>A | H3088Y | G | G | G | A | G | G | G | G | G | G | G |  | G | G | G | G | G | G | G | G | G | G |
|  | rs61743677 | T>C | K2930R | T | T | T | C | T | T | T | T | T | T | T |  | T | T | T | T | T | T | T | T | T | T |
|  | rs72731014 | T>C | T620A | T | T | T | C | T | C | T | C | T | C | C |  | T | C | C | C | T | T | C | C | C | C |
|  | rs55810732 | G>T | A614V | G | G | G | T | G | T | G | T | G | T | T |  | G | T | T | T | G | G | T | T | T | T |
|  | rs72731016 | G>C | Q593E | G | G | G | C | G | C | G | C | G | C | C |  | G | C | C | C | G | G | C | C | C | C |
|  | rs4696593 | G>C | S562R | G | G | G | G | G | G | C | G | G | G | G |  | G | G | G | G | G | G | G | G | G | G |
|  | rs79215995 | G>C | H940Q | G | G | G | G | G | G | G | G | C | G | G |  | G | G | G | G | G | G | G | G | G | G |
|  | rs61746132 | G>A | P3131L | G | G | G | G | G | G | G | G | G | G | G |  | G | G | G | G | G | G | G | G | G | A |
| **EFNA1** | rs4745 | A>T | D159V | T | T | T | T | T | T | T | A | A | T | T |  | T | T | T | T | T | T | T | T | A | T |
| **EFNA3** | rs17723260 | G>A | V190M | G | G | A | A | G | G | G | G | G | G | G |  | G | G | G | G | G | G | G | G | G | G |
| **EFNA4** | rs143886639 | C>A | P117T | C | A | C | C | C | C | C | C | C | C | C |  | C | C | C | C | C | C | C | C | C | C |
| **EPHA1** | rs6967117 | T>C | M900V | C | C | C | C | C | C | C | C | C | C | C |  | C | C | C | C | C | C | C | C | C | C |
|  | rs34372369 | G>A | P697L | A | G | G | G | G | G | G | G | G | G | G |  | G | A | G | G | G | G | A | G | A | G |
|  | rs4725617 | A>G | V160A | G | G | G | G | G | G | G | G | G | G | G |  | G | G | G | G | G | G | G | G | G | G |
|  | rs10952549 | G>T | L613M | G | G | G | G | T | G | G | G | G | G | G |  | G | G | G | G | G | G | G | G | G | G |
|  | rs11768549 | C>T | R492Q | C | C | C | C | C | C | C | C | C | T | C |  | C | C | C | C | C | C | C | C | C | C |
| **EPHA10** | rs6671088 | C>T | G749E | C | T | T | T | C | C | T | C | C | T | C |  | T | C | T | C | C | T | C | C | C | T |
|  | rs4653328 | A>T | F281I | A | T | T | A | A | A | T | A | T | T | T |  | T | A | A | A | T | T | T | T | A | A |
|  | rs11583463 | C>T | A955T | C | C | C | C | C | C | C | C | T | C | C |  | C | C | C | C | C | C | C | C | C | C |
|  | rs6670599 | C>T | R807Q | C | C | C | C | C | C | C | C | T | C | C |  | C | C | C | C | C | C | C | C | C | T |
|  | rs12405650 | C>T | V645I | C | C | C | C | C | C | C | C | T | C | C |  | C | C | C | C | C | C | C | C | C | T |
|  | rs17511304 | A>G | L629P | A | A | A | A | A | A | A | A | G | A | A |  | A | A | A | A | A | A | A | A | A | G |
| **EPHA2** | rs376937559 | G>A | S636L | G | G | G | G | A | G | G | G | G | G | G |  | G | G | G | G | G | G | G | G | G | G |
|  | rs11543934 | G>T | R350T | G | G | G | G | T | G | G | G | G | G | G |  | G | G | G | G | G | G | G | G | G | G |
|  | rs35903225 | C>T | R876H | C | C | C | C | C | C | C | C | C | C | C |  | C | T | C | C | C | C | C | C | C | C |
| **EPHA3** | rs17801309 | G>A | R914H | G | G | G | A | G | G | G | G | G | G | G |  | G | G | G | A | G | A | G | G | A | G |
| **EPHA6** | rs301948 | G>A | E346K | A | G | G | G | G | A | G | G | G | G | A |  | A | A | G | A | G | A | A | A | G | G |
|  | rs301949 | A>G | M346V | G | A | A | A | A | G | A | A | A | A | G |  | G | G | A | G | A | G | G | G | A | A |
|  | rs200518665 | G>A | V305I | G | G | G | G | G | A | G | G | G | G | G |  | G | G | G | G | G | G | G | G | G | G |
|  | rs200120931 | T>A | L990H | T | T | T | T | T | T | T | T | T | T | T |  | A | T | T | T | T | T | T | T | T | T |
| **EPHA7** | rs2278106 | G>A | P278S | G | G | G | A | G | G | G | G | G | G | G |  | G | G | A | G | G | G | G | G | G | G |
|  | rs2278107 | T>C | I138V | T | T | T | C | T | T | T | T | T | T | T |  | T | T | C | T | T | T | T | T | T | T |
| **EPHA8** | rs999765 | G>C | E612Q | G | G | G | C | G | G | G | G | G | G | C |  | G | G | G | G | G | G | G | G | C | G |
| **EPHB1** | rs202034365 | G>A | V562I | G | G | G | G | G | G | G | G | G | G | G |  | G | G | G | A | G | G | G | G | G | G |
| **EPHB6** | rs8177173 | G>A | G122S | G | A | G | G | G | G | G | G | G | G | G |  | G | G | G | G | A | G | G | G | G | G |
| **GFRA1** | rs8192662 | A>T | Y85N | A | A | A | A | T | A | A | A | A | A | A |  | A | A | A | A | T | A | A | A | A | A |
|  | rs2072276 | T>C | T366A | T | T | T | T | T | T | T | T | T | C | T |  | T | C | T | T | T | T | T | C | T | T |
| **NGF** | rs6330 | G>A | A35V | A | A | A | A | A | G | A | A | A | A | A |  | A | A | A | A | A | A | A | G | A | A |
|  | rs138175552 | G>A | R83C | G | G | G | G | G | A | G | G | G | G | G |  | G | G | G | G | G | G | G | G | G | G |
|  | rs11466111 | C>T | R80Q | C | C | C | C | C | C | C | C | C | C | C |  | C | T | C | C | C | C | C | T | C | C |
| **NRP1** | rs2228638 | C>T | V733I | C | C | C | T | C | C | C | C | C | T | C |  | C | C | C | C | C | C | C | C | C | T |
| **NRP2** | rs200483574 | insA | C901Stop | - | - | - | - | - | - | - | - | - | - | - |  | - | insA | - | - | - | - | - | - | - | - |
| **NTN3** | rs139072382 | G>A | D32N | G | G | G | G | G | G | G | G | G | G | A |  | G | G | G | G | G | G | G | G | G | G |
| **NTN4** | rs17288108 | A>G | Y205H | A | A | A | G | A | A | G | G | A | G | G |  | A | G | G | A | G | A | A | A | G | A |
|  | rs34114770 | G>A | R73G | G | G | G | G | A | G | G | G | G | G | G |  | G | G | G | G | G | G | G | G | G | G |
|  | rs34684875 | C>A | G25V | C | C | C | C | C | C | C | C | C | C | C |  | C | C | C | C | C | C | A | C | C | C |
| **NTNG2** | rs4962173 | A>G | T346A | G | G | G | G | G | G | G | G | G | G | G |  | G | G | G | G | G | G | G | G | G | G |
| **NTRK1** | rs1007211 | G>A | G18E | G | A | G | G | G | G | G | G | G | G | G |  | G | G | G | A | G | G | G | G | G | G |
|  | rs367836863 | G>A | G169R | G | G | A | G | G | G | G | G | G | G | G |  | G | G | G | G | G | G | G | G | G | G |
|  | rs35669708 | G>A | R780P | G | G | G | G | G | G | G | G | G | G | G |  | A | G | G | G | G | G | G | G | G | G |
|  | rs6336 | C>T | H604Y | C | C | C | C | C | C | C | C | C | C | C |  | C | C | C | C | C | C | C | T | C | C |
|  | rs6339 | G>T | G613V | G | G | G | G | G | G | G | G | G | G | G |  | G | G | G | G | G | G | G | T | G | G |
| **PCDHGA12** | rs78612001 | C>A | A559E | C | C | C | C | C | C | A | C | C | C | C |  | C | C | C | A | C | C | C | C | C | C |
| **PLAU** | rs2227564 | T>C | L141P | C | C | C | C | C | C | C | C | C | C | C |  | C | C | C | C | C | C | C | C | C | C |
| **PLAUR** | rs2302524 | T>C | K220R | T | T | T | T | C | T | T | T | T | C | T |  | T | T | T | C | T | C | T | T | C | T |
|  | rs4760 | A>G | L317P | A | A | A | A | A | G | A | A | A | G | G |  | A | A | A | G | A | A | A | A | A | G |
| **PLXNA1** | rs147504334 | A>G | S1635G | A | A | A | A | A | A | A | A | A | A | A |  | A | A | G | A | A | A | A | A | A | A |
| **PLXNA2** | rs4844658 | T>C | E369G | T | T | T | C | C | T | T | T | C | T | C |  | T | T | C | T | T | C | T | C | C | C |
|  | rs201718455 | A>G | V750A | A | A | A | A | A | G | A | A | A | A | A |  | A | A | A | A | A | A | A | A | A | A |
|  | rs34457681 | C>T | V1536M | C | C | C | C | C | C | C | T | C | C | C |  | T | C | C | C | C | T | C | C | C | C |
|  | rs17011882 | G>C | A805G | G | G | G | G | G | G | G | G | G | G | G |  | G | G | G | G | C | G | G | G | G | G |
| **PLXNA3** | rs5945430 | G>C | E863D | G | G | C | G | C | G | G | G | C | G | G |  | G | G | G | C | G | G | G | G | G | G |
|  | rs138319096 | C>T | S175L | C | C | C | T | C | C | C | C | C | C | C |  | C | C | C | C | C | C | C | C | C | C |
|  | rs139336954 | C>T | P653L | C | C | C | C | C | C | C | C | C | C | C |  | T | C | C | C | C | C | C | C | C | C |
| **PLXNA4** | rs741664 | T>C | M458V | C | T | C | C | C | C | C | C | T | C | C |  | C | C | C | C | T | C | T | C | C | C |
|  | rs62622406 | C>T | A891T | C | C | C | C | C | C | C | C | T | C | C |  | C | C | C | T | C | C | C | C | C | C |
|  | rs183271681 | G>A | T471M | G | G | G | G | G | G | G | G | G | G | A |  | G | G | G | G | G | G | G | G | G | G |
| **PLXNB2** | rs11547731 | T>C | I823V | C | C | T | T | C | C | C | C | C | T | C |  | C | T | C | C | T | C | C | C | C | C |
|  | rs79966207 | T>C | N759D | T | T | T | T | C | T | C | T | T | T | C |  | C | T | T | T | T | T | T | T | T | C |
|  | rs62621372 | C>T | V935I | C | C | C | C | C | C | C | C | C | C | C |  | T | C | C | C | C | C | C | C | C | C |
|  | rs28470336 | T>C | T812A | T | T | T | T | T | T | T | T | T | T | T |  | T | T | T | T | T | C | T | T | T | T |
| **PLXNB3** | rs2266879 | G>A | V598I | A | A | A | A | A | A | A | G | G | A | G |  | A | A | A | A | G | A | A | A | G | A |
|  | rs6643791 | G>C | E1156D | C | C | C | C | C | C | C | C | C | C | C |  | C | C | C | C | G | C | C | C | C | C |
|  | rs5987155 | T>C | M1558T | C | C | C | C | C | C | T | C | C | C | C |  | C | C | C | C | T | C | C | C | C | C |
| **PLXNC1** | rs75674989 | G>T | R614S | G | G | G | G | G | G | T | G | G | G | G |  | G | G | G | G | G | G | T | G | G | G |
|  | rs115651556 | G>A | R554Q | G | G | G | G | G | G | G | A | G | G | G |  | G | G | G | G | G | G | G | G | G | G |
| **PLXND1** | rs2713625 | C>T | S1542N | T | T | T | T | T | T | T | T | T | T | T |  | T | T | T | T | C | T | T | C | T | T |
|  | rs2625962 | T>C | H894R | C | C | C | C | C | C | C | C | C | C | C |  | C | C | C | C | C | C | C | C | C | C |
|  | rs2625973 | A>C | L1412V | A | C | A | C | C | A | A | A | A | C | A |  | A | A | C | A | A | A | C | A | C | C |
|  | rs2255703 | T>C | M870V | T | C | C | C | C | C | T | T | T | C | T |  | T | T | C | T | T | T | C | T | C | C |
|  | rs2285372 | G>A | P617S | G | G | G | G | G | A | G | G | G | G | G |  | G | G | G | G | G | G | G | G | G | G |
|  | rs2301572 | C>T | G531S | C | C | C | C | C | T | C | C | C | C | C |  | C | C | C | C | C | C | C | C | C | C |
|  | rs79870266 | C>G | G292A | C | C | C | C | C | C | C | C | C | C | C |  | C | C | G | C | C | C | C | C | C | C |
| **RET** | rs148935214 | C>T | S649L | C | C | C | C | C | C | C | C | C | C | C |  | C | C | C | C | C | C | C | T | C | C |
| **SEMA3A** | rs139295139 | T>C | N153S | T | T | T | T | T | T | T | T | C | T | T |  | T | T | T | T | T | T | T | T | T | T |
|  | rs147436181 | C>T | V435I | C | C | C | C | C | C | C | C | C | C | C |  | C | C | C | T | C | C | C | C | C | C |
|  | rs138952094 | G>A | T717I | G | G | G | G | G | G | G | G | G | G | G |  | G | G | G | G | G | G | G | G | A | G |
| **SEMA3B** | rs67324803 | C6>C7 | frameshift | C6 | C6 | C6 | C6 | C6 | C6 | C6 | C6 | C7 | C7 | C6 |  | C6 | C6 | C6 | C6 | C6 | C6 | C6 | C6 | C6 | C6 |
|  | rs2071203 | C>T | T415I | C | C | C | C | C | C | C | C | C | C | C |  | C | C | C | C | C | C | C | C | C | T |
| **SEMA3C** | rs140244551 | A>G | V321A | A | A | A | A | A | A | A | A | A | G | A |  | A | A | A | A | A | A | A | A | A | A |
| **SEMA3D** | rs7800072 | T>G | K701Q | T | G | G | G | G | T | T | T | T | T | T |  | G | T | G | G | G | T | T | G | G | T |
| **SEMA3E** | rs61729612 | C>G | R208P | C | G | C | G | C | G | G | C | C | G | C |  | G | C | C | C | C | C | C | G | C | C |
| **SEMA3G** | rs2016575 | T>C | M41V | T | C | C | C | C | T | T | C | C | C | C |  | T | C | C | C | C | C | C | C | C | C |
|  | rs200593686 | G>A | P692S | G | G | G | G | G | G | G | A | G | G | G |  | G | G | G | G | G | G | G | G | G | G |
|  | rs138988057 | G>A | A47V | G | G | G | G | G | G | G | G | G | G | G |  | G | G | A | G | G | G | G | G | G | G |
| **SEMA4B** | rs4932305 | G>A | M63I | A | G | A | A | G | A | A | G | A | G | A |  | G | G | A | G | G | G | G | G | A | G |
| **SEMA4D** | rs45464494 | C>T | G592D | T | C | C | C | C | C | T | C | C | C | T |  | C | C | C | C | C | T | T | C | T | T |
|  | rs62638726 | G>A | P703S | A | G | G | G | G | G | A | G | G | G | A |  | G | G | G | G | G | A | A | G | A | A |
|  | rs13295305 | C>T | A713T | C | C | C | C | C | C | C | C | C | C | C |  | T | T | T | T | C | T | C | T | C | T |
| **SEMA4F** | rs139676653 | G>A | V79I | G | G | G | G | G | G | G | A | G | G | G |  | G | G | G | G | G | G | G | G | G | G |
| **SEMA4G** | rs11591349 | A>T | D597V | T | A | A | T | A | T | T | T | T | T | A |  | A | T | A | A | T | T | T | T | A | A |
|  | rs11190780 | T>C | M612T | T | T | T | T | C | T | C | T | T | T | T |  | T | T | T | T | T | T | T | T | C | T |
|  | rs41291464 | G>A | A708H | G | G | G | G | G | G | G | G | G | G | A |  | G | A | G | G | G | G | G | G | G | G |
| **SEMA5A** | rs34563995 | T>C | S956G | T | C | T | T | T | T | T | T | T | T | T |  | T | T | T | T | T | T | T | T | T | T |
| **SEMA5B** | rs2303983 | T>C | D1028G | C | C | C | C | C | C | T | C | C | C | C |  | T | T | C | C | C | T | C | C | C | C |
|  | rs2276782 | A>T | V840D | T | T | T | T | T | T | A | T | T | T | T |  | T | T | T | T | T | T | T | T | T | A |
|  | rs2276774 | A>G | I220P | A | A | A | A | A | A | A | G | G | A | A |  | G | A | G | A | A | A | A | A | A | G |
|  | rs2276781 | A>G | M742T | A | A | A | A | A | A | A | A | G | A | A |  | A | A | A | A | A | A | A | G | A | A |
|  | rs2303982 | T>G | D1034G | T | T | T | T | T | T | T | T | T | T | T |  | T | G | T | T | T | T | T | G | G | T |
| **SEMA6A** | rs34966 | G>A | H518Y | G | A | G | G | G | G | G | G | G | G | G |  | G | G | G | A | G | G | G | G | G | G |
|  | rs17432496 | C>T | R559H | C | C | T | C | C | C | C | C | C | C | C |  | C | C | C | C | C | C | C | C | C | C |
|  | rs114860630 | C>T | G795S | C | C | C | C | C | C | C | C | C | C | C |  | C | C | C | C | C | T | C | C | C | C |
| **SEMA6C** | rs4971007 | T>G | T455P | G | G | G | G | G | G | G | G | G | G | G |  | G | G | G | T | G | T | G | G | G | G |
|  | rs75185260 | T>C | T68A | T | C | T | T | T | T | T | T | T | T | T |  | T | T | T | T | T | T | T | T | T | T |
| **SEMA6D** | rs532598 | G>A | S478A | A | G | G | A | G | A | G | A | G | A | A |  | G | A | A | G | A | A | A | G | A | A |
|  | rs146886309 | A>G | H698R | G | A | A | A | A | A | A | A | A | A | A |  | A | A | A | A | A | A | A | A | A | A |
| **SEMA7A** | rs741761 | T>C | G515H | C | T | T | T | T | T | T | T | T | T | T |  | T | T | T | T | T | T | T | T | T | T |
| **UNC5B** | rs34957097 | A>G | I242V | A | A | A | A | A | A | A | A | A | A | A |  | G | A | G | A | A | A | A | A | A | A |
|  | rs10509332 | G>A | A516T | G | G | G | G | G | G | G | G | G | G | G |  | G | A | G | G | G | G | G | G | G | G |
|  | rs138539665 | G>A | R175H | G | G | G | G | G | G | G | G | G | G | G |  | G | G | G | G | G | A | G | G | G | G |
| **UNC5C** | rs4699423 | A>G | S97R | G | A | A | A | A | A | A | A | A | A | A |  | A | A | A | A | A | A | A | A | A | A |
|  | rs2289043 | A>G | M721T | A | G | G | G | G | G | A | G | G | G | G |  | G | G | G | G | G | G | A | G | G | G |
|  | rs34585936 | C>T | A841T | C | C | C | C | C | C | C | T | C | T | C |  | C | C | C | C | C | C | C | C | C | C |
| **VEGFC** | rs55728985 | T>A | E47V | T | T | T | T | T | T | T | A | T | T | T |  | T | T | T | T | T | T | T | T | T | T |
|  | rs3062984 | TCAT>T | LS>delR | TCAT | TCAT | TCAT | TCAT | TCAT | TCAT | TCAT | TCAT | delR | TCAT | TCAT |  | TCAT | TCAT | TCAT | TCAT | TCAT | TCAT | TCAT | TCAT | TCAT | TCAT |
| **VLDLR** | rs144724569 | C>G | F484L | C | C | C | G | C | C | C | C | C | C | C |  | C | C | C | C | C | C | C | C | C | C |

**Supplementary Table 6.** The incidence of some genetic variants in brain morphogenic genes identified in "schizophrenia" and "depression" (MDD) groups in the Russian population (men and women). Contr - control, SCZ - Schizophrenia, MDD - major depression disorder.

| **Gene** | **SNP** | **Group** | **N** | **Var1** | **Var1/Var2** | **Var2** | **P** | **Allele, %** | | **Χ^2^** | **P** |
| --- | --- | --- | --- | --- | --- | --- | --- | --- | --- | --- | --- |
| ***BDNF*** | **rs6265 (C>T, V66M)** |  |  | **C/C** | **C/T** | **T/T** |  | **C** | **T** |  |  |
|  |  | Contr | 103 | 64 | 29 | 1 |  | 157 | 31 |  |  |
|  |  | SCZ | 102 | 77 | 19 | 5 | *0.0641* | 173 | 29 | *0.196* | *0.658* |
|  |  | MDD | 79 | 52 | 21 | 2 | *0.7469* | 125 | 25 | *0.0107* | *0.917* |
| ***CDH13*** | **rs4782724 (C>T, P55S)** |  |  | **C/C** | **C/T** | **T/T** |  | **C** | **T** |  |  |
|  |  | Contr | 103 | 2 | 9 | 92 |  | 13 | 193 |  |  |
|  |  | SCZ | 102 | 1 | 7 | 94 | *0.7770* | 9 | 195 | *0.402* | *0.526* |
|  |  | MDD | 79 | 2 | 8 | 69 | *0.7899* | 12 | 146 | *0.0735* | *0.786* |
| ***DCHS1*** | **rs4758443 (G>A, T1949M)** |  |  | **G/G** | **G/A** | **A/A** |  | **G** | **A** |  |  |
|  |  | Contr | 103 | 32 | 54 | 16 |  | 118 | 86 |  |  |
|  |  | SCZ | 102 | 46 | 46 | 10 | *0.1037* | 138 | 66 | *3.785* | *0.052* |
|  |  | MDD | 79 | 25 | 36 | 15 | *0.7095* | 86 | 66 | *0.017* | *0.896* |
| ***PLAU*** | **rs2227564 (T>C, L141P)** |  |  | **T/T** | **T/C** | **C/C** |  | **T** | **C** |  |  |
|  |  | Contr | 103 | 8 | 40 | 51 |  | 56 | 142 |  |  |
|  |  | SCZ | 102 | 4 | 36 | 61 | *0.3177* | 44 | 158 | *1.92* | *0.166* |
|  |  | MDD | 79 | 6 | 37 | 36 | *0.6791* | 49 | 109 | *0.197* | *0.657* |
| ***PLAUR*** | **rs4760 (A>G, L224P)** |  |  | **A/A** | **A/G** | **G/G** |  | **A** | **G** |  |  |
|  |  | Contr | 103 | 66 | 34 | 1 |  | 166 | 36 |  |  |
|  |  | SCZ | 102 | 58 | 39 | 5 | *0.1720* | 155 | 49 | *1.996* | *0.158* |
|  |  | MDD | 79 | 53 | 24 | 2 | *0.6780* | 130 | 28 | *0.013* | *0.909* |

* - Over 20% of the expected values in the contingency table are less than 5. The Chi-square test is inaccurate.

**Supplementary Table 7.** The incidence of some genetic variants in brain morphogenic genes identified in "schizophrenia-female" and "depression-female" (MDD) groups in the Russian population. Contr - control, SCZ - Schizophrenia, MDD - major depression disorder.

| **Gene** | **SNP** | **Group** | **N** | **Var1** | **Var1/Var2** | **Var2** | **P** | **Allele, %** | | **Χ^2^** | **P** |
| --- | --- | --- | --- | --- | --- | --- | --- | --- | --- | --- | --- |
| ***CDH3*** | **rs12923655 (A>C, T808P)** |  |  | **A/A** | **A/C** | **C/C** |  | **A** | **C** |  |  |
|  |  | Contr | 71 | 27 | 24 | 17 |  | 78 | 58 |  |  |
|  |  | SCZ | 47 | 15 | 18 | 14 | *0.7008* | 48 | 46 | 0.652 | 0.419 |
|  |  | MDD | 60 | 26 | 14 | 20 | *0.2919* | 66 | 54 | 0.0637 | 0.801 |
|  | **rs3114409 (A>C, R778S)** |  |  | **A/A** | **A/C** | **C/C** |  | **A** | **C** |  |  |
|  |  | Contr | 71 | 34 | 26 | 8 |  | 94 | 42 |  |  |
|  |  | SCZ | 47 | 23 | 19 | 5 | *1.0000* | 65 | 29 | 0.0196 | 0.889 |
|  |  | MDD | 60 | 38 | 19 | 3 | *0.2368* | 95 | 25 | 2.832 | 0.092 |
| ***CDH13*** | **rs4782724 (C>T, P55S)** |  |  | **C/C** | **C/T** | **T/T** |  | **C** | **T** |  |  |
|  |  | Contr | 71 | 1 | 7 | 63 |  | 9 | 133 |  |  |
|  |  | SCZ | 47 | 1 | 3 | 43 | *0.8732* | 5 | 89 | 0.00184 | 0.966 |
|  |  | MDD | 60 | 1 | 6 | 53 | *0.8852* | 8 | 112 | 0.0208 | 0.885 |
| ***DCHS1*** | **rs4758443 (G>A, T1949M)** |  |  | **G/G** | **G/A** | **A/A** |  | **G** | **A** |  |  |
|  |  | Contr | 71 | 20 | 38 | 12 |  | 78 | 62 |  |  |
|  |  | SCZ | 47 | 19 | 23 | 5 | *0.3619* | 61 | 33 | 1.603 | 0.206 |
|  |  | MDD | 60 | 22 | 25 | 11 | *0.4059* | 69 | 47 | 0.23 | 0.631 |
| ***DCHS2*** | **rs12500437 (G>T, P1342H)** |  |  | **G/G** | **G/T** | **T/T** |  | **G** | **T** |  |  |
|  |  | Contr | 71 | 1 | 5 | 62 |  | 7 | 129 |  |  |
|  |  | SCZ | 47 | 1 | 10 | 36 | *0.0647* | 12 | 82 | 3.311 | 0.069 |
|  |  | MDD | 60 | 0 | 10 | 49 | *0.1667* | 10 | 108 | 0.651 | 0.42 |
|  | **rs72731014 (T>C, T620A)** |  |  | **T/T** | **T/C** | **C/C** |  | **T** | **C** |  |  |
|  |  | Contr | 71 | 45 | 18 | 4 |  | 108 | 26 |  |  |
|  |  | SCZ | 47 | 23 | 22 | 2 | *0.0880* | 68 | 26 | 1.696 | 0.193 |
|  |  | MDD | 60 | 38 | 21 | 1 | *0.3596* | 97 | 23 | 0.0125 | 0.911 |
|  | **rs28561984 (C>T, E2050K)** |  |  | **C/C** | **C/T** | **T/T** |  | **C** | **T** |  |  |
|  |  | Contr | 71 | 49 | 20 | 2 |  | 118 | 24 |  |  |
|  |  | SCZ | 47 | 34 | 13 | 0 | *0.7721* | 81 | 13 | 0.205 | 0.651 |
|  |  | MDD | 60 | 37 | 22 | 1 | *0.5101* | 96 | 24 | 0.236 | 0.627 |
|  | **rs1352714 (T>C, N1352S)** |  |  | **T/T** | **T/C** | **C/C** |  | **T** | **C** |  |  |
|  |  | Contr | 71 | 2 | 3 | 63 |  | 7 | 129 |  |  |
|  |  | SCZ | 47 | 0 | 0 | 47 | *0.2163* | 0 | 94 | -* | -* |
|  |  | MDD | 60 | 1 | 1 | 58 | *0.7154* | 3 | 117 | -* | -* |
|  | **rs11935573 (G>A, S1660L)** |  |  | **G/G** | **G/A** | **A/A** |  | **G** | **A** |  |  |
|  |  | Contr | 71 | 15 | 43 | 10 |  | 73 | 63 |  |  |
|  |  | SCZ | 47 | 13 | 29 | 5 | *0.6979* | 55 | 39 | 0.349 | 0.555 |
|  |  | MDD | 60 | 17 | 35 | 6 | *0.5884* | 69 | 47 | 0.638 | 0.424 |
| ***PLAU*** | **rs2227564 (T>C, L141P)** |  |  | **T/T** | **T/C** | **C/C** |  | **T** | **C** |  |  |
|  |  | Contr | 71 | 5 | 30 | 33 |  | 40 | 96 |  |  |
|  |  | SCZ | 46 | 2 | 16 | 28 | *0.4557* | 20 | 72 | 1.294 | 0.255 |
|  |  | MDD | 60 | 6 | 29 | 25 | *0.6958* | 41 | 79 | 0.465 | 0.495 |
| ***PLAUR*** | **rs4760 (A>G, L224P)** |  |  | **A/A** | **A/G** | **G/G** |  | **A** | **G** |  |  |
|  |  | Contr | 71 | 41 | 27 | 1 |  | 109 | 29 |  |  |
|  |  | SCZ | 47 | 28 | 17 | 2 | *0.6620* | 73 | 21 | 0.00616 | 0.937 |
|  |  | MDD | 60 | 43 | 15 | 2 | *0.2165* | 101 | 19 | 0.821 | 0.365 |

* - Over 20% of the expected values in the contingency table are less than 5. The Chi-square test is inaccurate.

**Supplementary Table 8.** The incidence of some genetic variants in brain morphogenic genes identified in "schizophrenia-male" and "depression-male" (MDD) groups in the Russian population. Contr - control, SCZ - Schizophrenia, MDD - major depression disorder.

| **Gene** | **SNP** | **Group** | **N** | **Var1** | **Var1/Var2** | **Var2** | **P** | **Allele, %** | | **Χ^2^** | **P** |
| --- | --- | --- | --- | --- | --- | --- | --- | --- | --- | --- | --- |
| ***BDNF*** | **rs6265 (C>T, V66M)** |  |  | **C/C** | **C/T** | **T/T** |  | **C** | **T** |  |  |
|  |  | Contr | 32 | 19 | 11 | 1 |  | 49 | 13 |  |  |
|  |  | SCZ | 55 | 39 | 13 | 2 | *0.5452* | 91 | 17 | 0.424 | *0.515* |
|  |  | MDD | 19 | 13 | 5 | 0 | *0.8435* | 31 | 5 | 0.362 | *0.547* |
| ***CDH2*** | **rs1944294 (A>T, L21Stop)** |  |  | **A/A** | **A/T** | **T/T** |  | **A** | **T** |  |  |
|  |  | Contr | 32 | 24 | 8 | 0 |  | 56 | 8 |  |  |
|  |  | SCZ | 55 | 33 | 19 | 2 | *0.3754* | 85 | 23 | 1.551 | *0.213* |
|  |  | MDD | 19 | 16 | 3 | 0 | *0.5048* | 35 | 3 | -* | -* |
|  | **rs17445840 (C>T, A118T)** |  |  | **C/C** | **C/T** | **T/T** |  | **C** | **T** |  |  |
|  |  | Contr | 32 | 29 | 2 | 0 |  | 60 | 2 |  |  |
|  |  | SCZ | 55 | 49 | 6 | 0 | *0.7053* | 104 | 6 | -* | -* |
|  |  | MDD | 19 | 16 | 0 | 2 | *0.1264* | 32 | 4 | -* | -* |
| ***CDH13*** | **rs4782724 (C>T, P55S)** |  |  | **C/C** | **C/T** | **T/T** |  | **C** | **T** |  |  |
|  |  | Contr | 32 | 1 | 2 | 29 |  | 4 | 60 |  |  |
|  |  | SCZ | 55 | 0 | 4 | 51 | *0.4876* | 4 | 106 | -* | -* |
|  |  | MDD | 19 | 1 | 2 | 16 | *0.8179* | 4 | 34 | -* | -* |
| ***CDH23*** | **rs1227051 (G>A, A1575T)** |  |  | **G/G** | **G/A** | **A/A** |  | **G** | **A** |  |  |
|  |  | Contr | 32 | 1 | 8 | 22 |  | 10 | 52 |  |  |
|  |  | SCZ | 55 | 3 | 14 | 37 | *1.0000* | 20 | 88 | 0.034 | *0.854* |
|  |  | MDD | 19 | 1 | 5 | 13 | *1.0000* | 7 | 31 | 0.00048 | *0.982* |
|  | **rs10999947 (G>A, S496N)** |  |  | **G/G** | **G/A** | **A/A** |  | **G** | **A** |  |  |
|  |  | Contr | 32 | 17 | 14 | 1 |  | 48 | 16 |  |  |
|  |  | SCZ | 55 | 30 | 19 | 6 | *0.4156* | 79 | 31 | 0.0777 | *0.78* |
|  |  | MDD | 19 | 9 | 9 | 1 | *0.8925* | 27 | 11 | 0.0419 | *0.838* |
| ***DCHS1*** | **rs4758443 (G>A, T1949M)** |  |  | **G/G** | **G/A** | **A/A** |  | **G** | **A** |  |  |
|  |  | Contr | 32 | 12 | 16 | 4 |  | 40 | 24 |  |  |
|  |  | SCZ | 55 | 27 | 23 | 5 | *0.5895* | 77 | 33 | 0.721 | *0.396* |
|  |  | MDD | 19 | 3 | 11 | 4 | *0.2964* | 17 | 19 | 1.615 | *0.204* |
| ***PLAU*** | **rs2227564 (T>C, L141P)** |  |  | **T/T** | **T/C** | **C/C** |  | **T** | **C** |  |  |
|  |  | Contr | 32 | 3 | 10 | 18 |  | 16 | 46 |  |  |
|  |  | SCZ | 55 | 2 | 20 | 33 | *0.5344* | 24 | 86 | 0.165 | *0.684* |
|  |  | MDD | 19 | 0 | 8 | 11 | *0.4719* | 8 | 30 | 0.0895 | *0.765* |

* - Over 20% of the expected values in the contingency table are less than 5. The Chi-square test is inaccurate.
